# Dialect Variation Influences the Phonological and Lexical-Semantic Word Processing in Sentences. Electrophysiological Evidence from a Cross-Dialectal Comprehension Study

**DOI:** 10.3389/fpsyg.2016.00739

**Published:** 2016-05-27

**Authors:** Manuela Lanwermeyer, Karen Henrich, Marie J. Rocholl, Hanni T. Schnell, Alexander Werth, Joachim Herrgen, Jürgen E. Schmidt

**Affiliations:** ^1^Forschungszentrum Deutscher Sprachatlas, Philipps-Universität MarburgMarburg, Germany; ^2^Max Planck Institute for Empirical AestheticsFrankfurt am Main, Germany

**Keywords:** dialect perception, phonological variation, semantic processing, N200, N400, late positive component (LPC)

## Abstract

This event-related potential (ERP) study examines the influence of dialectal competence differences (merged vs. unmerged dialect group) on cross-dialectal comprehension between Southern German dialects. It focuses on the question as to whether certain dialect phonemes (/$\overset{⌢}{\text{oa}}$/, /$\overset{⌢}{\text{o}Ʊ}$/), which are attributed to different lexemes in two dialect areas (Central Bavarian, Bavarian-Alemannic transition zone) evoke increased neural costs during sentence processing. In this context, the phonological and semantic processing of lexemes is compared in three types of potentially problematic communication settings (misunderstanding, incomprehension, allophonic variation = potential comprehension). For this purpose, an oddball design including whole sentences was combined with a semantic rating task. Listeners from the unmerged Central Bavarian dialect area heard sentences including either native or non-native lexemes from the merged neighboring dialect. These had to be evaluated with regard to their context acceptability. The main difference between the lexemes can be attributed to the fact that they have different meanings in the respective dialect areas or are non-existent in the linguistic competence of the Central Bavarians. The results provide evidence for the fact that non-native lexemes containing the /$\overset{⌢}{\text{oa}}$/-diphthong lead to enhanced neural costs during sentence processing. The ERP results show a biphasic pattern (N2b/N400, LPC) for non-existent lexemes (incomprehension) as well as for semantically incongruous lexemes (misunderstanding), reflecting an early error detection mechanism and enhanced costs for semantic integration and evaluation. In contrast, allophonic /$\overset{⌢}{\text{o}Ʊ}$/ deviations show reduced negativities and no LPC, indexing an unproblematic categorization and evaluation process. In the light of these results, an observed change of /$\overset{⌢}{\text{oa}}$/ to /$\overset{⌢}{\text{o}Ʊ}$/ in the Bavarian-Alemannic transition zone can be interpreted as a facilitation strategy of cross-dialectal comprehension to reduce both misunderstandings as well as neural costs in processing, which might be interpreted as the initial trigger for this particular phoneme change.

## Introduction

### Phoneme change as a result of dialect contact

Despite the intensive preoccupation with the phenomenon of linguistic change, the question as to why linguistic units change and which factors influence this process is still a matter of debate. Several studies show that lexically irregular changes are primarily the result of dialect contact (cf. for example Trudgill, 1986; Wang and Lien, 1993; Schmidt and Herrgen, 2011)^1^. These changes result from the interference between systems and the interaction of speakers with different phonological competences.

In this context, one interesting phenomenon is the merger of a phonemic contrast in one dialect, which is still maintained in a related dialect. The expansion of unconditioned mergers can be explained by the close contact between merged and unmerged speech communities. For instance, the actuation of the low back merger in Pennsylvania is explained by a massive influx of foreign-born immigrants who had difficulties in acquiring the distinction between long and short /o/ in words like *cot* and *caught* due to their own reduced vowel systems. Thus, the expansion of the merger is the result of repeated misunderstandings of productions of one-phoneme-speakers by two-phoneme-speakers in face-to-face communication (cf. Herold, 1997; Labov, 2010). Overall, misunderstandings between groups of regional speakers are often motivated by differences in their linguistic competences (cf. Labov, 2010; Schmidt, 2010; Schmidt and Herrgen, 2011).

**Figure 1:**
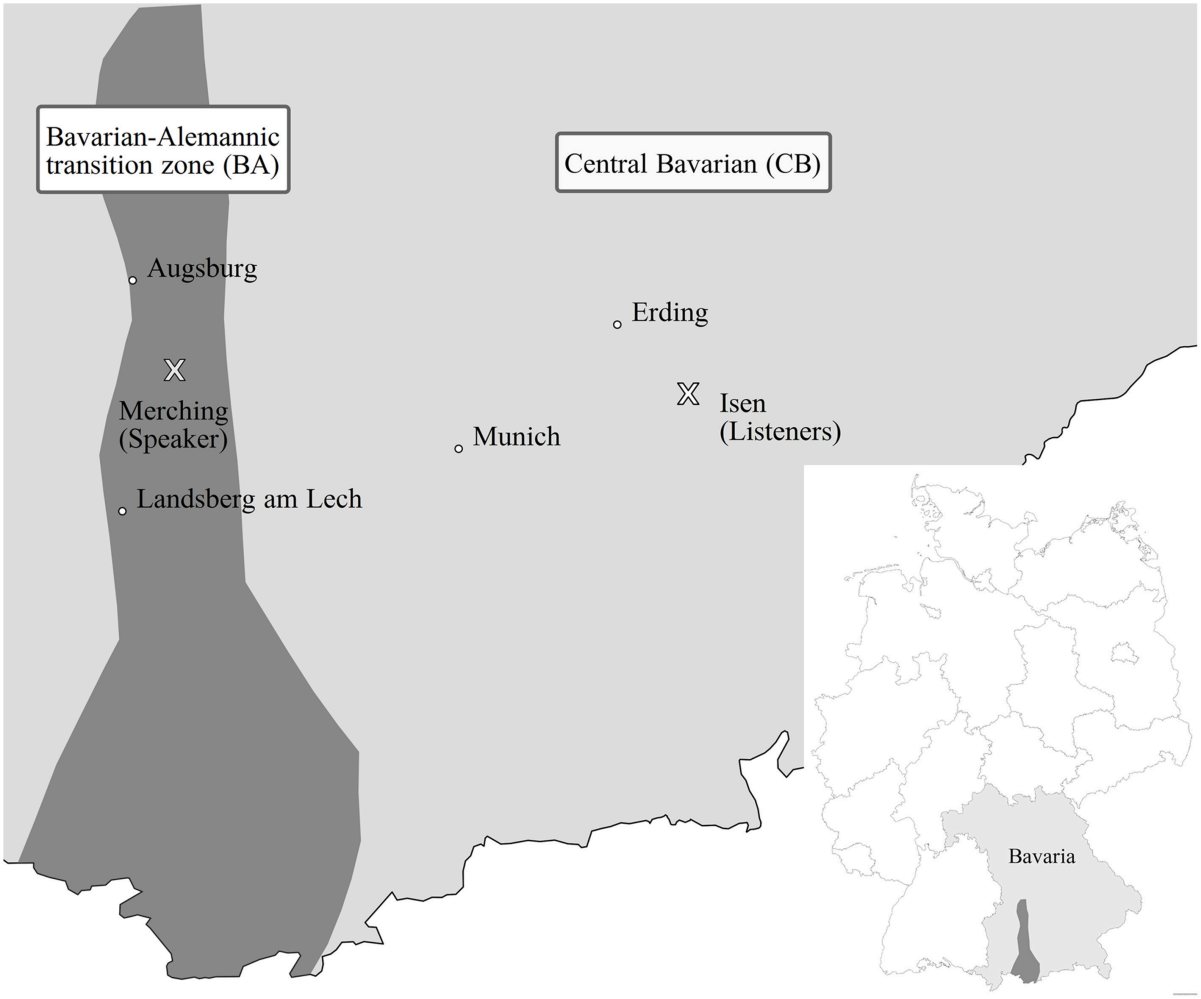
**The Bavarian-Alemannic transition zone (BA) and the Central Bavarian dialect area (CB) with × displaying the recording location (Merching) and experimentation location (Isen)**.

In order to investigate the relationship between cross-dialectal comprehension and phoneme change, it is useful to study similar varieties, which differ only in few phoneme contrasts so that the general understanding between the speaker groups is ensured. This particular setting can be found in two Southern German dialects. Therefore, the current study investigates the dialect /$\overset{⌢}{\text{oa}}$/-/$\overset{⌢}{\text{o}Ʊ}$/ contrast, which has developed differently in these areas. In the Central Bavarian dialect (henceforth CB), it is a stable contrast, while in the neighboring Bavarian-Alemannic transition zone (henceforth BA) only /$\overset{⌢}{\text{oa}}$/ occurs before obstruents, brought forth by a merger of Middle High German (MHG) *ô* and *ei* (see Figure 1 for the geographic location of the dialect areas). Interestingly, a phoneme change to either /$\overset{⌢}{\text{o}Ʊ}$/ or /oː/ can be observed in certain lexemes in BA, which is possibly due to the dialect contact with CB (Schmidt and Herrgen, 2011). Even if this development has been documented by production data, no perception study has tested this assumption so far. To investigate this gap in research, a study employing event-related potentials (ERPs) was conducted focusing on cross-dialectal comprehension between both of these dialect groups.

Our study focuses on the question whether the usage of dialect phonemes (/$\overset{⌢}{\text{oa}}$/, /$\overset{⌢}{\text{o}Ʊ}$/) which are attributed to different lexemes in the two contiguous dialect areas, leading to minimal pairs between these areas, evoke increased neural costs during sentence processing. If so, this would indicate difficulties in cross-dialectal comprehension. We are especially interested in semantic processing differences elicited by minimal phonological differences between different phoneme contact settings (misunderstanding, incomprehension, allophonic variation) in the form they can appear in everyday communication situations.

### The impact of regional variation on phoneme perception in online observation

Cross-dialectal comprehension is highly related to the capacity of listeners to deal with acoustic variability in pronunciation resulting from dialectal variation. A listener's ability to perceive different speech sounds as phonemes mainly depends on the phoneme inventory of his own native language (cf. e.g., Buchwald et al., 1994 concerning the discrimination between /r/ and /l/ in Japanese). Studies using the electroencephalography (EEG) technique provide evidence that this assumption can be adapted to non-native regional phonemic contrasts within a language, as well. For instance, Brunellière et al. (2011) compared the /e/-/ε/ contrast in word-final open syllables (e.g., /epe/ ‘sword’ vs. /epε/ ‘thick’) in merged and unmerged French speaker groups. They found processing differences concerning the cortial topographies, indicating that in contrast to unmerged speakers, merged speakers associate the two forms with only one semantic representation (homophones). These clear differences between the groups support the assumption that the access to lexical meaning in spoken word recognition heavily depends on the listeners' native regional accent. The influence of the native phoneme inventory on phoneme perception was also investigated by Conrey et al. (2005), who focus on mechanisms of semantic integration and phonological decision processes using the example of the /ɪ/ and /ε/ merger before nasal consonants in American English (the so-called *pin*-*pen* merger). The results show that in contrast to the unmerged group neither behavioral nor neural differences could be detected in the merged group. In contrast to previous studies, these results suggest that the different groups process the stimuli differently at a conscious, decisional level.

Previous studies dealing with dialect contrasts mainly used the Mismatch Negativity (MMN) component to examine vowel discrimination (cf. Brunellière et al., 2009, 2011; Scharinger et al., 2011). The MMN is a fronto-central negative component, usually peaking at 150–250 ms from change onset, when infrequent deviations (deviant stimuli) occur among frequently repeated sound patterns (standard stimuli) in a passive oddball design. The MMN is elicited regardless of the participant's direction of attention and thus reflects an automatic, pre-attentive response to any change in auditory stimulation. A basic prerequisite for MMN elicitation is the creation of a short-term memory trace in the auditory cortex, i.e., a representation of the repetitive standard stimulus. The MMN is the reflection of a discrimination process as the representation is violated by an infrequent deviant, indicating that the deviant is found to be incongruent with the memory representation of the preceding series of standard stimuli (cf. Näätänen et al., 2007). Using cross-linguistic oddball designs, several studies could establish language-specific memory traces for phonemes. The MMN deflection is increased when the deviant is a vowel category in the subject's native language in contrast to non-native vowel categories (Näätänen et al., 1997). Moreover, Kazanina et al. (2006) investigated the [t]-[d] contrast, which is mapped onto distinct phoneme categories in Russian, while it is an allophonic contrast in Korean. An MMNm was only elicited for the Russian listeners, indicating a rapid separation of these sounds into two categories, while the Korean listeners do not show any immediate sensitivity to the contrast. Furthermore, the MMN is also modulated by dialectal categories. Miglietta et al. (2013) compared the phonemic contrast [e]-[i] to the allophonic variation [ε]-[e] present in the Tricase dialect located in Southern Italy and found—in contrast to Kazanina et al. (2006)—an MMN response for both conditions. However, the latency of the phonemic condition was significantly earlier, pointing to a facilitated short-term memory trace formation in contrast to the allophonic condition.

In sum, these studies show that the electrophysiological investigation of allophonic and phonemic dialect contrasts is quite promising, since phonological and lexical processing stages are highly influenced by the listeners' regional accent.

### The present ERP study

In Southern Germany, the Bavarian (including CB) and the Alemannic dialect area adjoin each other. Between both areas, a transition zone (BA) is located, in which phonological forms of both dialects interact with each other (cf. Wiesinger, 1983).

The investigated /$\overset{⌢}{\text{oa}}$/-/$\overset{⌢}{\text{o}Ʊ}$/ contrast distinguishes BA and CB since it is a stable contrast in CB, while in BA /$\overset{⌢}{\text{oa}}$/ occurs exclusively. Thus, the crucial point is how the respective phonemes have been assigned to lexemes in the dialect areas (see Supplementary Material for a precise diachronic description). For instance, /r$\overset{⌢}{\text{oa}}$s$\underset{ˈ}{\text{n}}$/ means ‘roses’ and ‘journeys’ in BA, while in CB it only corresponds to ‘journeys’. Moreover, /ʃtr$\overset{⌢}{\text{oa}}$/ means ‘straw’ in BA, while in CB it is pronounced as /ʃtr$\overset{⌢}{\text{o}Ʊ}$/.

Thus, the differences in the speakers' competences lead to different form-meaning-associations in the dialect areas. As a result, they might evoke different kinds of dialect-related communication difficulties when lexemes containing the /$\overset{⌢}{\text{oa}}$/-phoneme traced back to MHG *ô* are used by speakers of BA. Three types of potential communication difficulties are reflected in our experimental conditions:

1. On the one hand, there might occur misunderstandings when a lexeme has different meanings in the two dialect areas. The usage of such lexemes in cross-dialectal communication leads to faulty decoding by the listeners. For example, the BA lexeme /r$\overset{⌢}{\text{oa}}$s$\underset{ˈ}{\text{n}}$/ for ‘roses’ means ‘journeys’ in CB.
2. On the other hand, there might arise incomprehension, since many BA words containing the /$\overset{⌢}{\text{oa}}$/-diphthong do not exist in CB. In these cases, the listeners do not have a lexical entry for the lexeme and cannot decode it. For example, the BA lexeme /ʃtr$\overset{⌢}{\text{oa}}$/ ‘straw’ is not part of the CB speakers' competence.
3. A further differentiation between the dialects relevant for the present study affects the pronunciation of MHG *ô* before nasals (e.g., *Lohn* ‘wage’, *Bohne* ‘bean’). Among other variants, one common pronunciation is /$\overset{⌢}{\text{o}Ʊ}$/ in BA and /oː/ in CB. In cross-dialectal communication, it seems likely that articulatory very similar contrasts which are not integrated in the change of meaning between the dialects do not lead to erroneous decoding (cf., the BA variant /l$\overset{⌢}{õ\tilde{Ʊ}}$/ versus the CB /lõː/ for ‘wage’).

An ERP study using an oddball design was conducted in order to study the effect of the different phoneme to lexeme assignments on cross-dialectal comprehension caused by the merger of MHG *ô* and MHG *ei* in BA (condition 1 and 2) in contrast to a probable pure allophonic contrast (condition 3).

In a typical oddball design, isolated phonemes, syllables or meaningful words are presented to participants while they watch a silent film. However, since in everyday communication listeners are faced with words embedded in complex sentences, we were interested in vowel perception during auditory sentence processing in order to investigate a more natural setting. This way, the influence of phonological differences on lexical-semantic processes should be made ascertainable.

It is however doubtful whether sentences can constitute an equally invariant acoustic context as isolated syllables or words against which deviants are normally compared. So far, only few studies have adapted the oddball design to questions of phonemic and semantic processes during sentence comprehension (e.g., Menning et al., 2005; Boulenger et al., 2011; Bendixen et al., 2014). Their results indeed demonstrate the sensitivity of the MMN to complex linguistic material and suggest that during natural speech processing, the brain rapidly extracts phonetic information from the continuous signal and forms memory traces in the auditory cortex. Thus, it seems that memory traces can also develop for complex sentences, which include large-scale details about phonetic features of the speech signal.

Following Bendixen et al. (2014), an experimental design combining a classic oddball paradigm and a semantic rating task was developed for the current study. In contrast to Bendixen et al. (2014) as well as Boulenger et al. (2011), the material used in this study also included semantic violations that might involve higher levels of processing such as semantic contextual integration. Effects of semantic integration are indicated by a rather late negativity peaking around 400 ms after stimulus onset in the EEG. The N400 is distributed primarily over centro-parietal sites and is typically elicited by sentence-final words that are semantically anomalous or of low cloze probability (cf. Kutas and Hillyard, 1980, 1984; Connolly and Phillips, 1994; Lau et al., 2008; Kutas and Federmeier, 2011). Thus, it displays the violation of predictions and expectations built up by the preceding sentence context since predictable words are easier to access from memory and it requires more resources to process an implausible or infrequent continuation (cf. Lau et al., 2008). In the design of Boulenger et al. (2011), late ERP effects in the N400 time window were found indicating the involvement of semantic integration mechanisms. Thus, an oddball design including a task seems very promising to address the interaction between early acoustic processes and late mechanisms of semantic integration.

In the current study, a member of the N200 family (MMN, N2b) might be evoked as in the comparable study of Bendixen et al. (2014) for the conditions misunderstanding and incomprehension. However, due to the embedded semantic violations, which form an important difference to that study, we expect to also find an N400 in the misunderstanding condition. In contrast, we expect the fewest costs in semantic and lexical processing for the potential allophonic variation (condition 3) since the deviant might be categorized as a potential, allophonic form of the standard.

## Materials and methods

In the current study, listeners' perception of four different lexemes is investigated during sentence processing. The following three conditions reflect the special phoneme contact between the dialects of BA and CB (see Table 1). The experiment was conducted in CB, subsequently the terms ‘standard’ and ‘deviant’ are used with regard to the Central Bavarian dialect. The Central Bavarian lexemes served as the standard (2/3), while the Bavarian-Alemannic lexemes form the deviants (1/3).

1. Condition 1: Misunderstanding
  Whereas in BA the (former) homophonic lexeme /r$\overset{⌢}{\text{oa}}$s$\underset{ˈ}{\text{n}}$/ signifies both ‘roses’ and ‘journeys’, in CB it only bears the meaning ‘journeys’. When speakers from both areas communicate with one another, the usage of /r$\overset{⌢}{\text{oa}}$s$\underset{ˈ}{\text{n}}$/ ‘roses’ could lead to misunderstandings because it is always understood as ‘journeys’.
  - All sentences presented prime for the meaning ‘roses’. The Central Bavarian variant /r$\overset{⌢}{\text{o}Ʊ}$s$\underset{ˈ}{\text{n}}$/ ‘roses’ is infrequently interrupted by the Bavarian-Alemannic variant /r$\overset{⌢}{\text{oa}}$s$\underset{ˈ}{\text{n}}$/, which means ‘journeys’ in Central Bavarian.
         e.g., *Was im Garten viel Pflege braucht, sind Rosen*. ‘That which needs much care in the garden, are roses’
  - All sentences presented prime for the meaning ‘journeys’. The Central Bavarian variant /r$\overset{⌢}{\text{oa}}$s$\underset{ˈ}{\text{n}}$/ ‘journeys’ is infrequently interrupted by the lexeme /r$\overset{⌢}{\text{o}Ʊ}$s$\underset{ˈ}{\text{n}}$/ ‘roses’.
         e.g., *Wofür er seinen Koffer packt, sind Reisen*. ‘That for which he is packing his suitcase, are journeys’
2. Condition 2: Incomprehension
  Whereas in BA the MHG lexeme *lôs* ‘sow’ is pronounced like /l$\overset{⌢}{\text{oa}}$s/, in CB the dialectal lexeme is /l$\overset{⌢}{\text{o}Ʊ}$s/. In regional communication, the usage of /l$\overset{⌢}{\text{oa}}$s/ may lead to incomprehension because this form is not part of the phonological competence of speakers from CB. Therefore, the lexical access fails.
  Half of the sentences prime for the meaning ‘sow’, half are neutral sentences. The Central Bavarian variant /l$\overset{⌢}{\text{o}Ʊ}$s/ is infrequently interrupted by the Bavarian-Alemannic variant /l$\overset{⌢}{\text{oa}}$s/.
       priming: e.g., *Was die kleinen Ferkel säugt, ist die Lous*. ‘That which is nursing the little piglets, is the sow'
       neutral: e.g., *Was er ihr genau beschreibt, ist die Lous*. ‘What he is describing to her exactly, is the sow'
3. Condition 3: Potential Comprehension
  Whereas in BA ‘wage’ is pronounced like /l$\overset{⌢}{õ\tilde{Ʊ}}$/, in CB the variant is /lõː/. Although the form is non-native to the listeners of CB, it is likely that it does not lead to erroneous decoding.
  Half of the sentences prime for the meaning ‘wage’, half are neutral sentences. The Bavarian variant /lõː/ is sporadically interrupted by the Bavarian-Alemannic variant /l$\overset{⌢}{õ\tilde{Ʊ}}$/.
       priming: e.g., *Was man am Monatsende bekommt, ist der Lohn*. ‘That which you receive at the end of the month, is the wage'
       neutral: e.g., *Worüber sie beim Treffen reden, ist der Lohn*. ‘That which they are talking about at the meeting, is the wage'

**Table 1:** **Experimental conditions**.

**Condition**	**Standard**		**Deviant**	
1a (misunderstanding)	/r$\overset{⌢}{\text{o}Ʊ}$s$\underset{ˈ}{\text{n}}$/	‘roses’	/r$\overset{⌢}{\text{oa}}$s$\underset{ˈ}{\text{n}}$/	‘journeys’
1b (misunderstanding)	/r$\overset{⌢}{\text{oa}}$s$\underset{ˈ}{\text{n}}$/	‘journeys’	/r$\overset{⌢}{\text{o}Ʊ}$s$\underset{ˈ}{\text{n}}$/	‘roses’
2 (incomprehension)	/l$\overset{⌢}{\text{o}Ʊ}$s/	‘sow’	/l$\overset{⌢}{\text{oa}}$s/	pseudo-word
3 (potential comprehension)	/lõː/	‘wage’	/l$\overset{⌢}{õ\tilde{Ʊ}}$/	potential allophonic form

### Pretests

A pool of German sentences ending in *Rosen* ‘roses’ (77), *Reisen* ‘journeys’ (104), *Lohn* ‘wage’ (85) or *Muttersau* ‘sow (female pig)' (75) was developed using standard language vocabulary and syntax. All sentences followed the same global structure of topicalized relative clauses with the critical item in sentence final position (e.g., *Was wild in der Hecke blüht, sind Rosen*. ‘That which is blossoming wildly in the hedge, are roses’). The sentences were designed to create a semantic expectation of the critical items. In addition, neutral sentences were created, which were integrated into conditions 2 and 3, in which the lexical meaning of the sentence-final lexemes does not differ, in order to keep the participants' attention. Using exclusively well-matched priming sentences would have been too invariant for the semantic rating task, so this was counterbalanced by adding neutral sentence contexts.

To guarantee that the sentences were classified as either priming or neutral context sentences, the high and low cloze probability of the sentences was surveyed within an online rating procedure. The full sentences were presented visually on a computer screen and participants had to evaluate on a scale from 1 to 7 whether the sentence-final word fit the context, with 1 indicating that the respective word did not fit at all and 7 indicating that the word fit very well (task 1). In a second task, participants had to decide whether the critical item fit better, worse or equally well into the sentence in comparison to other possible options (task 2). If the participants stated that other words fit better into the context, they were asked to write them down (task 3). All sentences from all conditions were mixed up randomly into equal parts and separated into 8 groups, so that each participant had to judge approximately 60 sentences. Altogether, 78 speakers of the standard variety participated in the rating task (54 women, mean age 32.03 (SD 12.51)). In the subsequent steps, only sentences which were evaluated as very suitable regarding the sentence-final items were selected for priming conditions, reflected by the mean of >6 respectively >5.5 for *Muttersau*. In contrast, for neutral context conditions, moderately well-judged sentences were selected, reflected by a mean of 1.8–4.9 (*Muttersau*) respectively 2.9–5.5 (*Lohn*). This rating procedure resulted in 35 sentences for each condition, in total 210 sentences.

### Stimuli

All 210 sentences were recorded several times by a male native speaker (year of birth 1963) who was born and raised in the Bavarian-Alemannic transition zone (Merching). He adapted the sentences to the dialect lexically and phonologically and produced the critical items in two variants each—his own and the Central Bavarian one. During the recordings, a natural pronunciation and a normal speech rate was ensured, as well as a comparable realization of the native and non-native lexemes with regard to their intensity and pitch. All stimuli were digitally recorded with a sampling rate of 44.1 kHz and a 16 bit (mono) sample size, using an electret microphone (Sony ECM-MS957).

For each condition, the best 30 sentences were selected from the recorded auditory material, leading to 180 sentences in total. Furthermore, 10 critical tokens of each item were chosen due to their F1 and F2 values being as similar as possible (see Table 2 for mean values). This acoustic variability was chosen in order to create a more natural speech perception and memory trace for the standard condition. This point is essential as it has been demonstrated that a higher and hence more natural variability in standard items allows for a more reliable abstraction or trace form from the different acoustic stimuli (cf. Phillips et al., 2000; Scharinger et al., 2011). Finally, four speakers from CB, who did not participate in the experiment, were asked to listen to the sentences to ensure that they are generally acceptable and comprehensible.

**Table 2:** **Phonetic values of the critical items (means)**.

**Critical item**	**Stimulus length [ms]**	**Vowel length [ms]**	**F1 [Hz]**		**F2 [Hz]**	
			20%	80%	20%	80%
/r$\overset{⌢}{\text{o}Ʊ}$s$\underset{ˈ}{\text{n}}$/	656	150	451.2	329.2	1114.7	1024.8
/r$\overset{⌢}{\text{oa}}$s$\underset{ˈ}{\text{n}}$/	633	147	378.4	505.5	807.1	1278.8
/l$\overset{⌢}{\text{o}Ʊ}$s/	628	118	398.7	320.0	1023.0	1042.1
/l$\overset{⌢}{\text{oa}}$s/	625	152	356.8	468.9	928.8	1238.5
/lõː/	527	186	348.4	335.7	736.1	704.2
/l$\overset{⌢}{õ\tilde{Ʊ}}$/	527	209	467.5	321.7	1154.1	759.0

The selected sentences were cross-spliced in order to use the same carrier sentence for standard and deviant conditions. To avoid different context inferences, a defined pause of 100 ms was inserted in front of the verb preceding the critical stimulus. In total, the sentences have an average length of 2.4 s. The dynamic range was manipulated in order to create a consistent sound because of the acoustic variation between and within sentences as a result of splicing. The pitch, duration or formants were not manipulated. Finally, all of the chosen items were controlled for and normalized in intensity. All of the adjustments were carried out using the software Adobe Audition CS6 (version 5.0.2).

### Procedure

During the experiment, participants were seated comfortably in a dimly lit and quiet room. A computer screen was placed in front of them. Participants were instructed to listen to the auditorily presented sentences and to evaluate on a four-point-scale how well the sentence-final word fit the sentence context after the offset of each sentence. For each word pair of the misunderstanding condition, 180 sentences (30 prime sentences, presented four times = 120 primes; 30 deviant sentences presented twice = 60 deviants) were presented, distributed over 2 blocks containing 90 sentences each. For the word pairs of the incomprehension and potential comprehension conditions, 360 sentences (30 prime sentences, presented four times = 120 primes; 30 deviant sentences, presented twice = 60 deviants / 30 neutral sentences, presented four times = 120 neutral sentences; 30 deviant sentences, presented twice = 60 neutral deviants) in total, were distributed over 4 blocks containing 90 sentences each. All blocks of one word pair were presented directly following each other with only short breaks in between. The two word pairs of the misunderstanding condition did not directly follow each other in the presentation. In total, 1080 sentences were presented in 12 blocks consisting of 90 sentences each (60 standards / primes, 30 deviants), with each block of approximately 7 min duration. Between separate blocks, participants were offered a short break to rest their eyes. In order to avoid sequence effects, the block order was varied across participants.

Before the experiment started, the participants completed a short practice phase to ensure that the given task and further instructions regarding eye blink phases were understood. Thereafter, the first experimental block started with the request to the participant to click any key to begin the experiment to ensure the participants' full attention when each block started. Each trial began with the presentation of a fixation cross in the center of the computer screen for 500 ms. A stimulus embedded in a carrier sentence was played via two loudspeakers while the fixation cross remained displayed on the screen to minimize eye movements. After the offset of each sentence, the fixation cross was replaced by a question mark which gave the signal for the participants to rate how well the sentence-final word fitted the sentence context as accurately and as quickly as possible by pressing one of four buttons within maximally 2000 ms. The assignment of buttons to four possible answers (very well, rather well, rather badly, very badly) was counterbalanced across participants. During the question mark phase, participants were allowed to blink and rest their eyes. 1000 ms after each response or time-out, the next trial started with an upcoming fixation cross. All procedures were performed in compliance with relevant laws and institutional guidelines.

### Participants

Twenty (13 women; mean age 44.5 (SD 4.87), age range 34–53) right-handed monolingual native speakers of German with normal or corrected-to-normal vision participated in the experiment. None of the participants had hearing deficits. All participants were born and raised in Isen (located in CB) and still live there. Their dialect competence was tested via a dialect pre-test. All participants gave their informed consent to this study and privacy rights were thoroughly obeyed. Each participant received monetary compensation for taking part in the study.

### ERP recording and data processing

An electroencephalogram (EEG) was recorded from 26 Ag/AgCl electrodes, mounted on an elastic cap (EasyCap), according to the 10–20 system (F7, F3, Fz, F4, F8, FC5, FC1, FCz, FC2, FC6, T7, C3, Cz, C4, T8, CP5, CP1, CPz, CP2, CP6, P7, P3, Pz, P4, P8, POz) with a *BrainVision* (Brain Products GmbH) amplifier. The C2 electrode served as ground electrode, the reference electrode was placed at the tip of the nose. Two further electrodes were placed at the left and right mastoid sites. To measure the electrooculogram (EOG), two electrodes were placed below and above the left eye and two further electrodes laterally to the outer canthi of both eyes in order to control for horizontal and vertical eye movements. All electrode impedances were kept below 5 kΩ. EEG and EOG were recorded continuously with a sampling rate of 500 Hz and filtered offline with a 0.3–20 Hz bandpass filter. This filter setting was chosen in order to remove slow drifts from the signal and the 20 Hz low pass filter was chosen following previous ERP studies investigating natural speech processing (cf. Menning et al., 2005; Partanen et al., 2011). Then, EEG recordings were re-referenced offline to the linked mastoids.

Prior to data analysis, all individual EEG recordings were scanned for artifacts from body movements or eye blinks. All artifacts exceeding an amplitude of 40 microvolt were automatically removed from the data set. A subsequent manual inspection of all single-trial waveforms scanned for further artifacts in all EEG channels. Data sets with more than 25% artifacts within one condition were excluded from further analyses. As a result of these inspections, the data set of 1 participant (1 male) had to be excluded from the misunderstanding condition and the potential comprehension condition; 2 data sets (1 male) had to be excluded from analysis for the incomprehension condition. These data sets were also excluded from the respective behavioral data analyses (see Supplementary Material for exact numbers of rejections). From the overall data set of the remaining participants, 5.6% of the misunderstanding condition stimuli, 6.0% of the incomprehension condition stimuli and 5.0% of the potential comprehension condition stimuli were excluded from analysis.

### Data analyses

The arithmetical mean of all responses for each condition was calculated by allocating a numerical value to each of the four possible response levels: 1 ≙ very well, 2 ≙ rather well, 3 ≙ rather badly, and 4 ≙ very badly. The arithmetical means were analyzed with an ANOVA separately for each condition pair, the respective factors are therefore presented in the results section of each condition (see Sections Behavioral data Condition 1: Misunderstanding–Behavioral data Condition 3: Potential comprehension). Further analyses of comparisons of each word pair were conducted using the Wilcoxon signed-rank test with a Bonferroni correction for the *p*-values.

In order to prevent movement artifacts, the evaluation response was given with a delay after the offset of the sentence. Due to this temporal distance between the perception of each critical item and the response, the measured reaction times for each evaluation response are not meaningful and not reported here.

For the EEG data, a multifactorial repeated-measures ANOVA was calculated with the factors condition (standard vs. deviant) and region [frontal (F3, FZ, F4), central (C3, CZ, C4), and parietal (P3, PZ, P4)]. Averages were calculated from onset of each sentence-final word up to 1000 ms thereafter for the misunderstanding condition and up to 900 ms thereafter for the two other conditions, with a baseline of 100 ms preceding the onset. The analysis was conducted with consecutive epochs of 50 ms from 0 ms up to 900 ms and 1000 ms respectively. Moreover, time windows for each paired comparison were also chosen based on hypotheses taken from the literature with similar experimental set-ups (Domahs et al., 2009; Boulenger et al., 2011; Bendixen et al., 2014) and were adjusted on the basis of visual inspection of the grand average curves. For effects with more than one degree of freedom, Huynh and Feldt (1976) corrections were applied to the *p*-values.

## Results

### Condition 1: misunderstanding

#### Behavioral data

The ANOVA for the misunderstanding condition with the factors diphthong (/r$\overset{⌢}{\text{o}Ʊ}$s$\underset{ˈ}{\text{n}}$/ ‘roses’ vs. /r$\overset{⌢}{\text{oa}}$s$\underset{ˈ}{\text{n}}$/ ‘journeys’) and expectancy (priming fulfilled vs. deviant) revealed only a main effect for the factor expectancy [*F*_(1, 18)_ = 605.67, *p* = 0.000], but not for the factor diphthong [*F*_(1, 18)_ = 0.56, *p* > 0.05], i.e., the diphthong itself did not have a significant influence on the participants' evaluation but only the compliance or non-compliance with the built-up expectancy due to the priming sentence context. Further analyses support this finding as they show that both correct priming conditions primed equally well regardless of the diphthong [/r$\overset{⌢}{\text{o}Ʊ}$s$\underset{ˈ}{\text{n}}$/ ‘roses’: mean 1.12 (SD 0.20) vs. /r$\overset{⌢}{\text{oa}}$s$\underset{ˈ}{\text{n}}$/ ‘journeys’: mean 1.15 (SD 0.17); *Z*_(18)_ = −1.01, *p* > 0.05] while the non-compliant deviants were evaluated as significantly less acceptable than the correct sentence-final words in the priming condition sentences [on a scale from 1 = acceptable to 4 = unacceptable; prime /r$\overset{⌢}{\text{o}Ʊ}$s$\underset{ˈ}{\text{n}}$/ ‘roses’ mean 1.12 (SD 0.20) vs. deviant /r$\overset{⌢}{\text{oa}}$s$\underset{ˈ}{\text{n}}$/ ‘journeys’: mean 3.72 (SD 0.79); *Z*_(18)_ = −3.79, *p* = 0.000 / prime /r$\overset{⌢}{\text{oa}}$s$\underset{ˈ}{\text{n}}$/ ‘journeys’: mean 1.15 (SD 0.17) vs. deviant /r$\overset{⌢}{\text{o}Ʊ}$s$\underset{ˈ}{\text{n}}$/ ‘roses’ mean 3.90 (SD 0.08); *Z*_(18)_ = −3.82, *p* = 0.000].

#### ERP data

**Figure 2:**
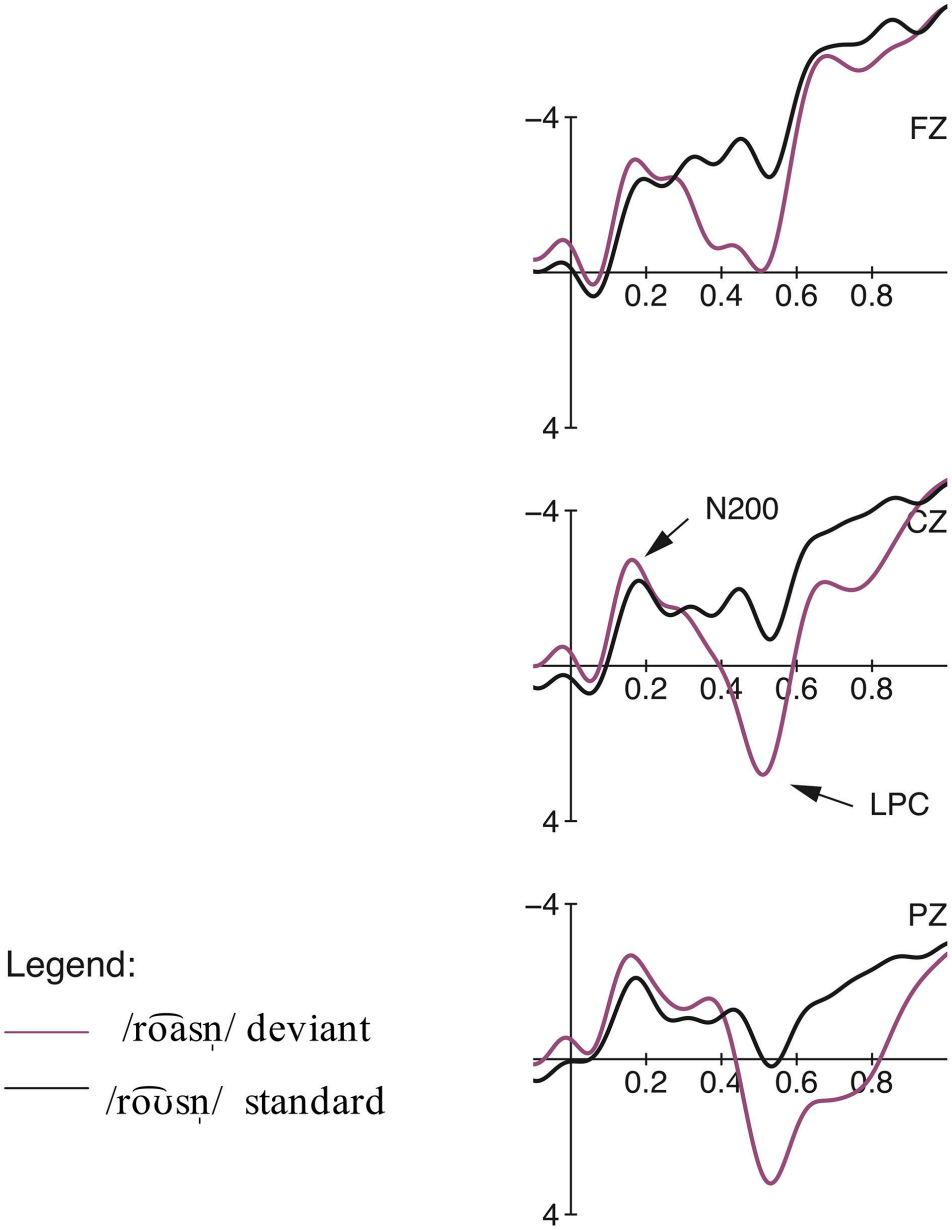
**Condition 1a: Misunderstanding: Grand averages of event-related potentials obtained for the deviant condition /r$\overset{⌢}{\text{oa}}$s$\underset{ˈ}{\text{n}}$/ and priming standard condition /r$\overset{⌢}{\text{o}Ʊ}$s$\underset{ˈ}{\text{n}}$/ measured from 100 ms prior the word onset up to 1000 ms**.

The comparison of the standard /r$\overset{⌢}{\text{o}Ʊ}$s$\underset{ˈ}{\text{n}}$/ ‘roses’ and the deviant /r$\overset{⌢}{\text{oa}}$s$\underset{ˈ}{\text{n}}$/ ‘journeys’ elicited an early negativity effect in the time window from 100 to 200 ms, followed by a late positivity effect from 400 to 900 ms (cf. Figures 2, **4**). The calculation of a repeated measures ANOVA revealed a significant main effect for the factor Expectancy (priming fulfilled vs. deviant) [*F*_(1, 18)_ = 11.52, *p* = 0.003, η^2^*p* = 0.05] but no significant interaction between the two factors Expectancy and region in the early time window [*F*_(2, 36)_ < 1, *p* > 0.05, η^2^*p* = 0.00]. In the later time window, the statistical analysis showed a main effect for both conditions [Expectancy: *F*_(1, 18)_ = 26.35, *p* = 0.000, η^2^*p* = 0.07; region: *F*_(2, 36)_ = 28.08, *p* = 0.000, η^2^*p* = 0.23] as well as a significant interaction between them [*F*_(2, 36)_ = 15.49, *p* = 0.000, η^2^*p* = 0.01]. The *post-hoc* analysis of this interaction by region revealed a stronger occurrence of the late positive component in the centro-parietal regions [frontal: *F*_(1, 18)_ = 7.37, *p* < 0.05, η^2^*p* = 0.01; central: *F*_(1, 18)_ = 26.55, *p* = 0.000, η^2^*p* = 0.12; parietal: *F*_(1, 18)_ = 44.45, *p* = 0.000, η^2^*p* = 0.28].

**Figure 3:**
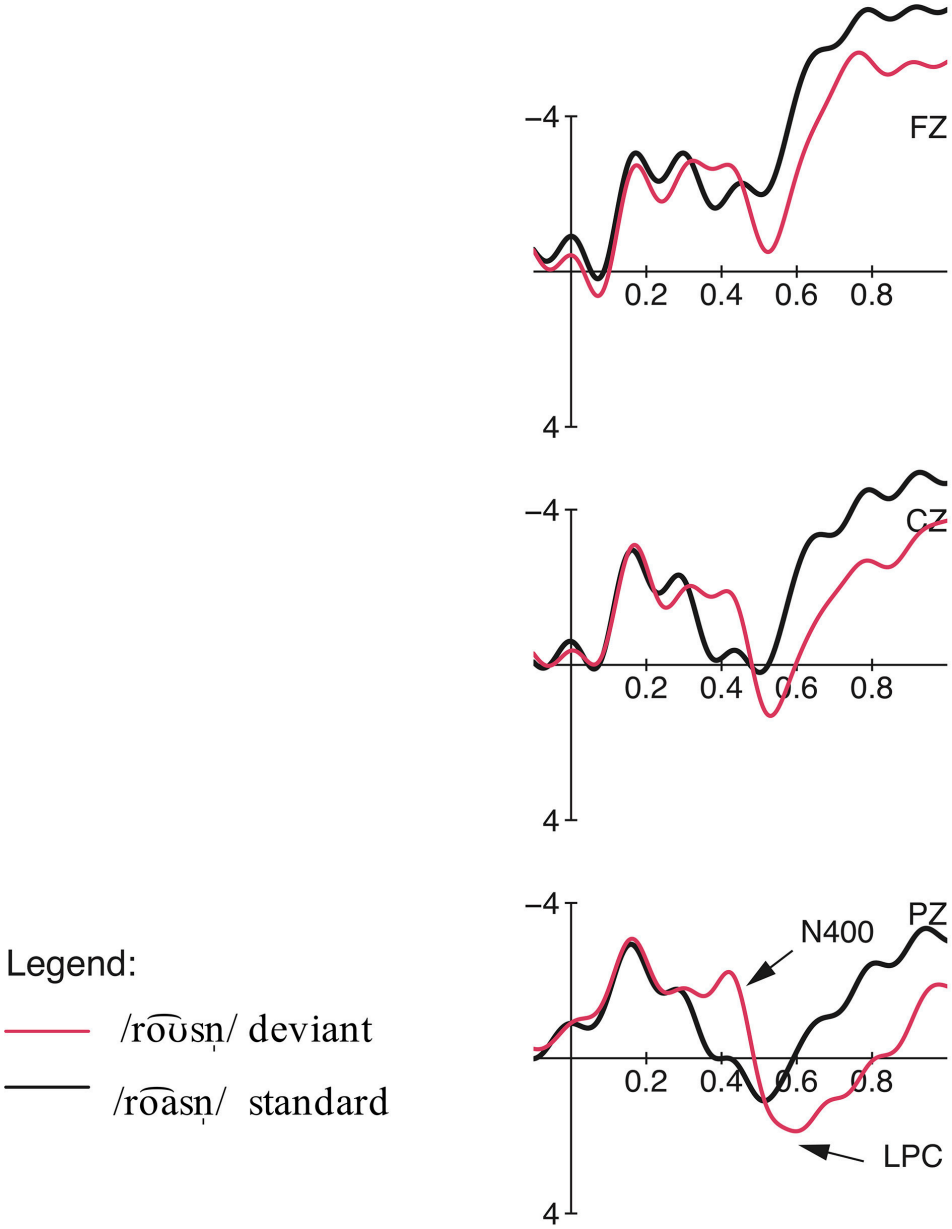
**Condition 1b: Misunderstanding: Grand averages of event-related potentials obtained for the deviant condition /r$\overset{⌢}{\text{o}Ʊ}$s$\underset{ˈ}{\text{n}}$/ and priming standard condition /r$\overset{⌢}{\text{oa}}$s$\underset{ˈ}{\text{n}}$/ measured from 100 ms prior the word onset up to 1000 ms**.

**Figure 4:**
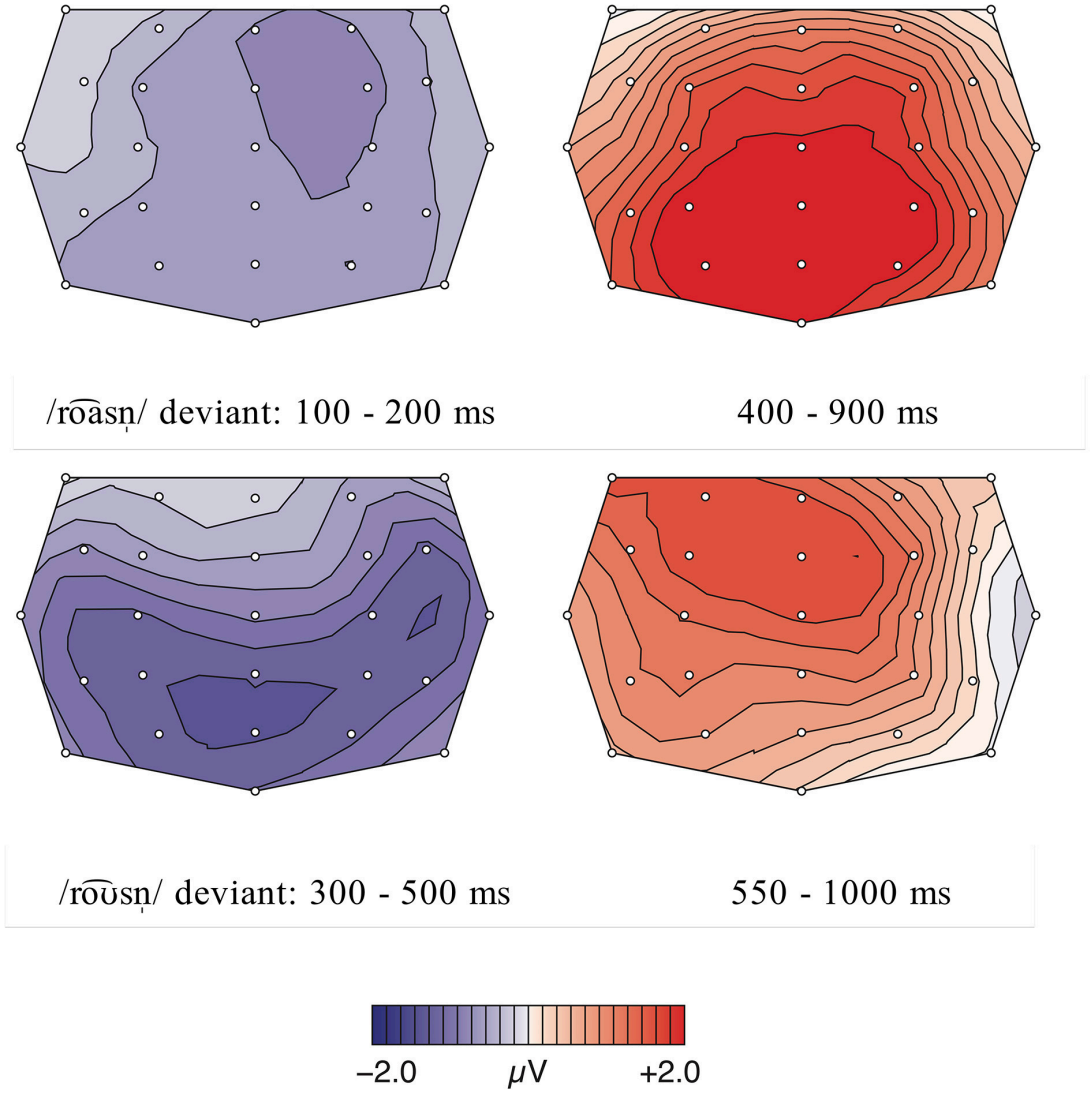
**(Top)** row: Topographic difference maps for the deviant condition /r$\overset{⌢}{\text{oa}}$s$\underset{ˈ}{\text{n}}$/ and priming standard condition /r$\overset{⌢}{\text{o}Ʊ}$s$\underset{ˈ}{\text{n}}$/ for the two significant time windows 100–200 ms and 400–900 ms. **(Lower)** row: Topographic difference maps for the deviant condition /r$\overset{⌢}{\text{o}Ʊ}$s$\underset{ˈ}{\text{n}}$/ and priming standard condition /r$\overset{⌢}{\text{oa}}$s$\underset{ˈ}{\text{n}}$/ for the two significant time windows 300–500 ms and 550–1000 ms.

For the comparison of the standard /r$\overset{⌢}{\text{oa}}$s$\underset{ˈ}{\text{n}}$/ ‘journeys’ and the deviant /r$\overset{⌢}{\text{o}Ʊ}$s$\underset{ˈ}{\text{n}}$/ ‘roses’ (see Figures 3, 4), the ANOVA showed no significant effects in the early negativity time window (100–200 ms) but a significant negativity effect was elicited by the deviant condition in a later time window from 300 to 500 ms [Expectancy: *F*_(1, 18)_ = 9.19, *p* = 0.007, η^2^*p* = 0.04; region: *F*_(2, 36)_ = 7.47, *p* = 0.012, η^2^*p* = 0.07]. The significant interaction between the factors Expectancy and region [*F*_(2, 36)_ = 5.37, *p* = 0.028, η^2^*p* = 0.01] resolved by region revealed a stronger occurrence of the early negativity in the centro-parietal region [frontal: *F*_(1, 18)_ = 1.33, *p* > 0.05, η^2^*p* = 0.00; central: *F*_(1, 18)_ = 10.53, *p* < 0.01, η^2^*p* = 0.08; parietal: *F*_(1, 18)_ = 14.96, *p* < 0.01, η^2^*p* = 0.13]. In the time window from 550 to 1000 ms, a positivity effect was elicited by the deviant condition [Expectancy: *F*_(1, 18)_ = 22.22, *p* = 0.000, η^2^*p* = 0.04; region: *F*_(2, 36)_ = 20.19, *p* = 0.000, η^2^*p* = 0.21] but there was no significant interaction between the two factors [*F*_(2, 36)_ = 2.66, *p* > 0.05, η^2^*p* = 0.00].

#### Discussion

In the misunderstanding condition, the deviant r$\overset{⌢}{\text{o}Ʊ}$s$\underset{ˈ}{\text{n}}$_/r$\overset{⌢}{\text{oa}}$s$\underset{ˈ}{\text{n}}$_ elicited a negativity effect in the latency range between 300 and 500 ms. Due to its latency as well as its centro-parietal scalp distribution, which is typical for the context-dependent N400 effect (cf. Lau et al., 2008; Kutas and Federmeier, 2011), we interpret this negativity as an N400 reflecting the semantic mismatch between the expected continuation of the sentence and the perceived input. The semantic priming of ‘journeys’ builds up an expectation for the correct word form /r$\overset{⌢}{\text{oa}}$s$\underset{ˈ}{\text{n}}$/. The semantically incongruous word /r$\overset{⌢}{\text{o}Ʊ}$s$\underset{ˈ}{\text{n}}$/ leads to a strong semantic mismatch between the context-based information held in working memory and the unfitting item. The N400 effect indexes an increased integration difficulty of the (incongruous) critical item with the prior sentence context (cf. Kutas and Hillyard, 1980; Brown and Hagoort, 1993; Kutas and Federmeier, 2000; Lau et al., 2009). Moreover, the N400 amplitude is modulated by the ease of accessing information from long-term memory (cf. Kutas and Federmeier, 2000; Lau et al., 2009). Due to the priming context, a congruous ending is pre-activated which is then disrupted by the incongruous sentence-final word. This disruption leads to higher processing costs, displayed by the pronounced N400 amplitude. The semantic mismatch between expectancy and perceived input is also reflected by the behavioral data, since sentences with unexpected final lexeme /r$\overset{⌢}{\text{o}Ʊ}$s$\underset{ˈ}{\text{n}}$/ were evaluated significantly worse than the sentences ending with the predictable word /r$\overset{⌢}{\text{oa}}$s$\underset{ˈ}{\text{n}}$/.

Interestingly, in the reversed condition our results show an earlier negativity effect between 100 and 200 ms for the deviant r$\overset{⌢}{\text{oa}}$s$\underset{ˈ}{\text{n}}$_/r$\overset{⌢}{\text{o}Ʊ}$s$\underset{ˈ}{\text{n}}$_. In previous studies, early negativities in similar time windows have been interpreted as detections of sudden changes in acoustic features of speech sounds embedded in sentences and have been classified as members of the N200 family (cf. Boulenger et al., 2011 for reversed speech; Bendixen et al., 2014 for omissions). The N200 component is distinguished into three sub-components (N2a, N2b, N2c) and is typically evoked between 180 and 325 ms respectively 100 and 200 ms (N2a) (cf. Patel and Azzam, 2005). Beside differences in latency and topography, early negativity effects are primarily separated on the basis of their sensitivity to varying task conditions. While the MMN (N2a) reflects pre-attentive passive change detections, the N2b is elicited by task relevant physical mismatches and thus requires attention to the stimuli. Thus, both components index different stages of mismatch detection (cf. Ritter et al., 1984; Pritchard et al., 1991; Folstein and Van Petten, 2008). Bendixen et al. (2014) support the component's dependency on active listening and its reflection of conscious error and mismatch detection, as well. In line with this perspective, we interpret the early negativity effect found for r$\overset{⌢}{\text{oa}}$s$\underset{ˈ}{\text{n}}$_/r$\overset{⌢}{\text{o}Ʊ}$s$\underset{ˈ}{\text{n}}$_ as an instance of the N2b, a reflection of a general rule-governed error detection mechanism. Since participants' attention was directed explicitly to the critical items due to the rating task, the N200 effect reflects an active discrimination and classification process, elicited by the deviation from a mentally-stored expectation of the standard stimulus (cf. Patel and Azzam, 2005), which more generally reflects the recognition of deviations and violations in regular structures (cf. Bohn et al., 2013; Henrich et al., 2014 for rhythmic irregularities). Thus, the semantic priming of ‘roses’ builds up an expectation for the correct word form /r$\overset{⌢}{\text{o}Ʊ}$s$\underset{ˈ}{\text{n}}$/. The deviation /r$\overset{⌢}{\text{oa}}$s$\underset{ˈ}{\text{n}}$/ is perceived as being different from the activated standard stimulus /r$\overset{⌢}{\text{o}Ʊ}$s$\underset{ˈ}{\text{n}}$/ and thus not fitting the activated memory trace.

Furthermore, late positive components (LPC) were elicited for r$\overset{⌢}{\text{o}Ʊ}$s$\underset{ˈ}{\text{n}}$_/r$\overset{⌢}{\text{oa}}$s$\underset{ˈ}{\text{n}}$_ (550–1000 ms) and r$\overset{⌢}{\text{oa}}$s$\underset{ˈ}{\text{n}}$_/r$\overset{⌢}{\text{o}Ʊ}$s$\underset{ˈ}{\text{n}}$_ (400–900 ms). We interpret these positivity effects as members of the P300 family, reflecting the evaluation process related to the given task requirements (Bentin et al., 1999; Knaus et al., 2007; Roehm et al., 2007; Domahs et al., 2008, 2009; Bohn et al., 2013; Henrich et al., 2014). Recall that participants had to perform a semantic rating task, i.e., their attention was directed consciously toward the linguistic material. Thus, the elicited positivity reflects the match or mismatch between the expected and encountered word form, i.e., the comparison of the critical stimulus with the expectation built-up by the memory trace of the standard form and—in priming contexts—the semantic information from the sentence context. The P300 is a positive deflection evoked by meaningful, task-relevant stimuli only when the subjects' attention is required for the task (cf. Picton, 1992). The P300 can be situated in processes of categorization, decision making, and context updating (cf. Coulson et al., 1998). Furthermore, the LPC may reflect a reanalysis process with regard to the semantic correctness of the presented sentences (cf. Domahs et al., 2008; Henrich et al., 2014). A deviating word form in sentence-final position requires a reanalysis and reevaluation of the previously built-up structure because the repetitively presented standard and the priming sentence context build up high expectations for a certain form. In this respect, the amplitude of the LPC is additionally modulated by the degree of the required reanalysis process, i.e., by the degree of deviation between what was expected and what was encountered.

### Condition 2: incomprehension

#### Behavioral data

The ANOVA for the incomprehension condition with the factors word type (correct word /l$\overset{⌢}{\text{o}Ʊ}$s/ ‘sow’ vs. pseudo-word /l$\overset{⌢}{\text{oa}}$s/) and sentence context (priming context vs. neutral context) showed main effects for both factors [word type: *F*_(1, 17)_ = 6.06, *p* = 0.025; sentence context: *F*_(1, 17)_ = 15.14, *p* = 0.001] but no significant interaction between the two factors [*F*_(1, 17)_ < 1, *p* > 0.05]. These results confirm that structures with the diphthong /$\overset{⌢}{\text{oa}}$/ were detected as non-existent pseudo-words and evaluated as significantly less acceptable than real words with the diphthong /$\overset{⌢}{\text{o}Ʊ}$/. A closer look at the arithmetical means shows that pseudo-words were apparently evaluated as less acceptable than real words when the priming sentence context built up expectancies which word should end the sentence. However, due to a high dispersion of the standard deviation, this difference is not statistically significant [correct word /l$\overset{⌢}{\text{o}Ʊ}$s/, mean 1.74 (SD 0.95) vs. pseudo-word /l$\overset{⌢}{\text{oa}}$s/, mean 2.72 (SD 1.31); *Z*_(17)_ = −1.68, *p* > 0.05]. In the neutral sentence contexts, pseudo-words were significantly rejected more often as unacceptable sentence-final words in comparison to the correct word form [correct word /l$\overset{⌢}{\text{o}Ʊ}$s/, mean 2.41 (SD 0.74) vs. pseudo-word /l$\overset{⌢}{\text{oa}}$s/, mean 3.07 (SD 0.91); *Z*_(17)_ = −2.33, *p* = 0.020]. The paired contrast of correct word forms depending on the embedded sentence context showed that the correct word form was evaluated as more acceptable in the priming sentence context [priming /l$\overset{⌢}{\text{o}Ʊ}$s/, mean 1.74 (SD 0.95) vs. neutral /l$\overset{⌢}{\text{o}Ʊ}$s/, mean 2.41 (SD 0.74); *Z*_(17)_ = −2.24, *p* = 0.025]. This shows that the sentence context had a significant influence on the lexical categorization process, i.e., real words were identified as such more easily when the sentence context gave specific cues and helped in building up expectations toward the incoming speech signal.

#### ERP data

**Figure 5:**
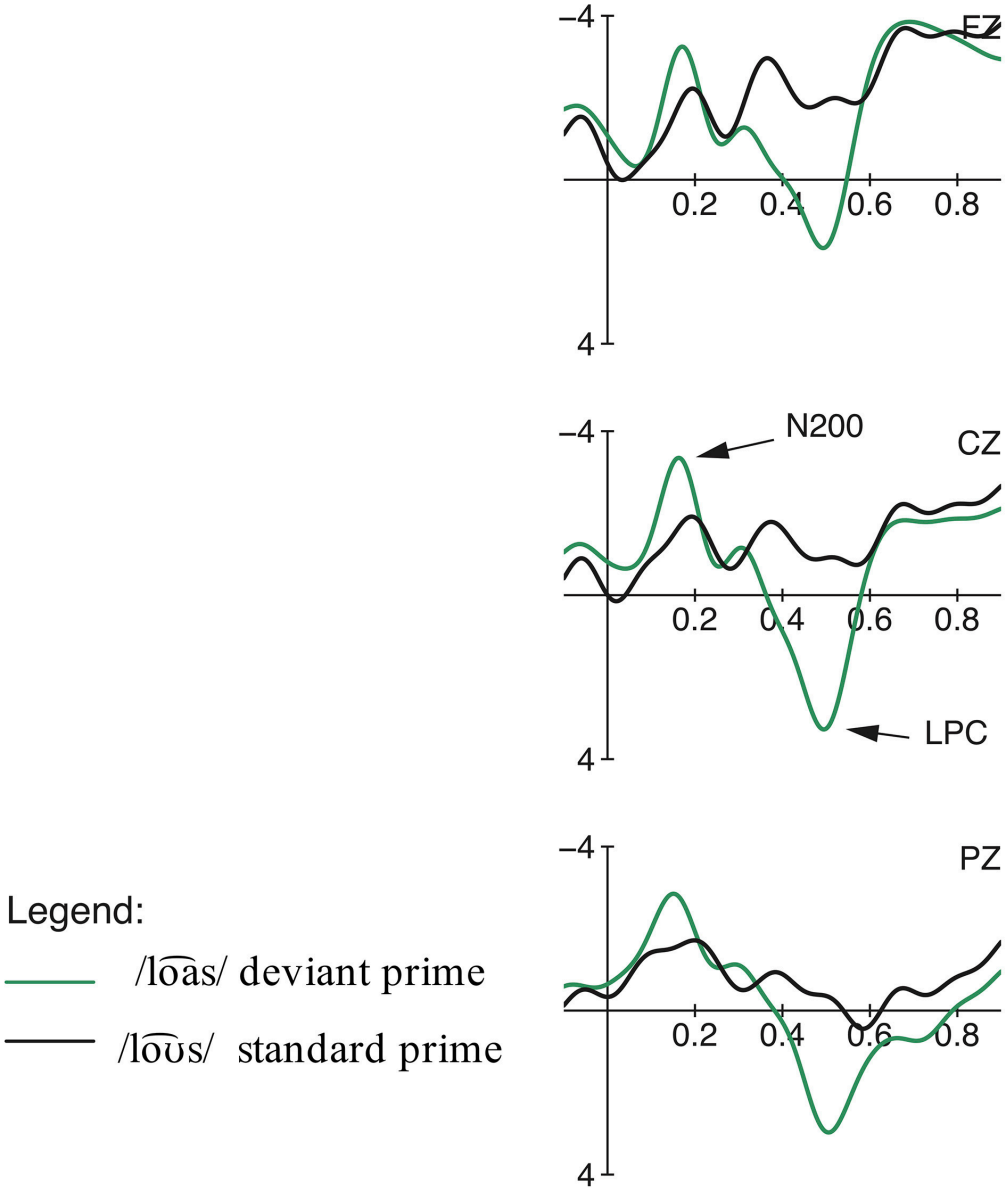
**Condition 2: Incomprehension, priming context: Grand averages of event-related potentials obtained for the deviant condition /l$\overset{⌢}{\text{oa}}$s/ and control standard condition /l$\overset{⌢}{\text{o}Ʊ}$s/ measured from 100 ms prior the word onset up to 900 ms**.

**Figure 6:**
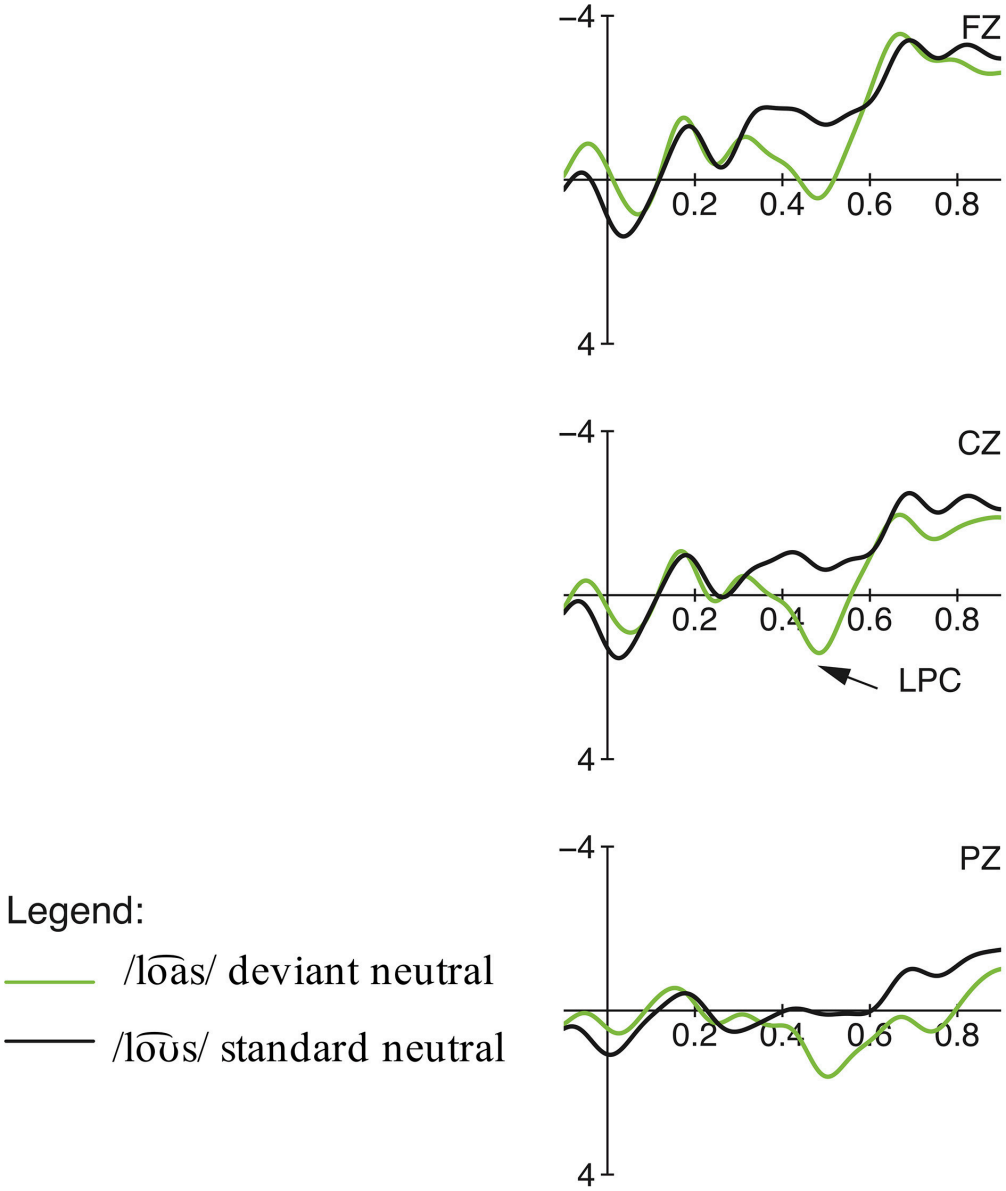
**Condition 2: Incomprehension, neutral context: Grand averages of event-related potentials obtained for the deviant condition /l$\overset{⌢}{\text{oa}}$s/ and control standard condition /l$\overset{⌢}{\text{o}Ʊ}$s/ measured from 100 ms prior the word onset up to 900 ms**.

**Figure 7:**
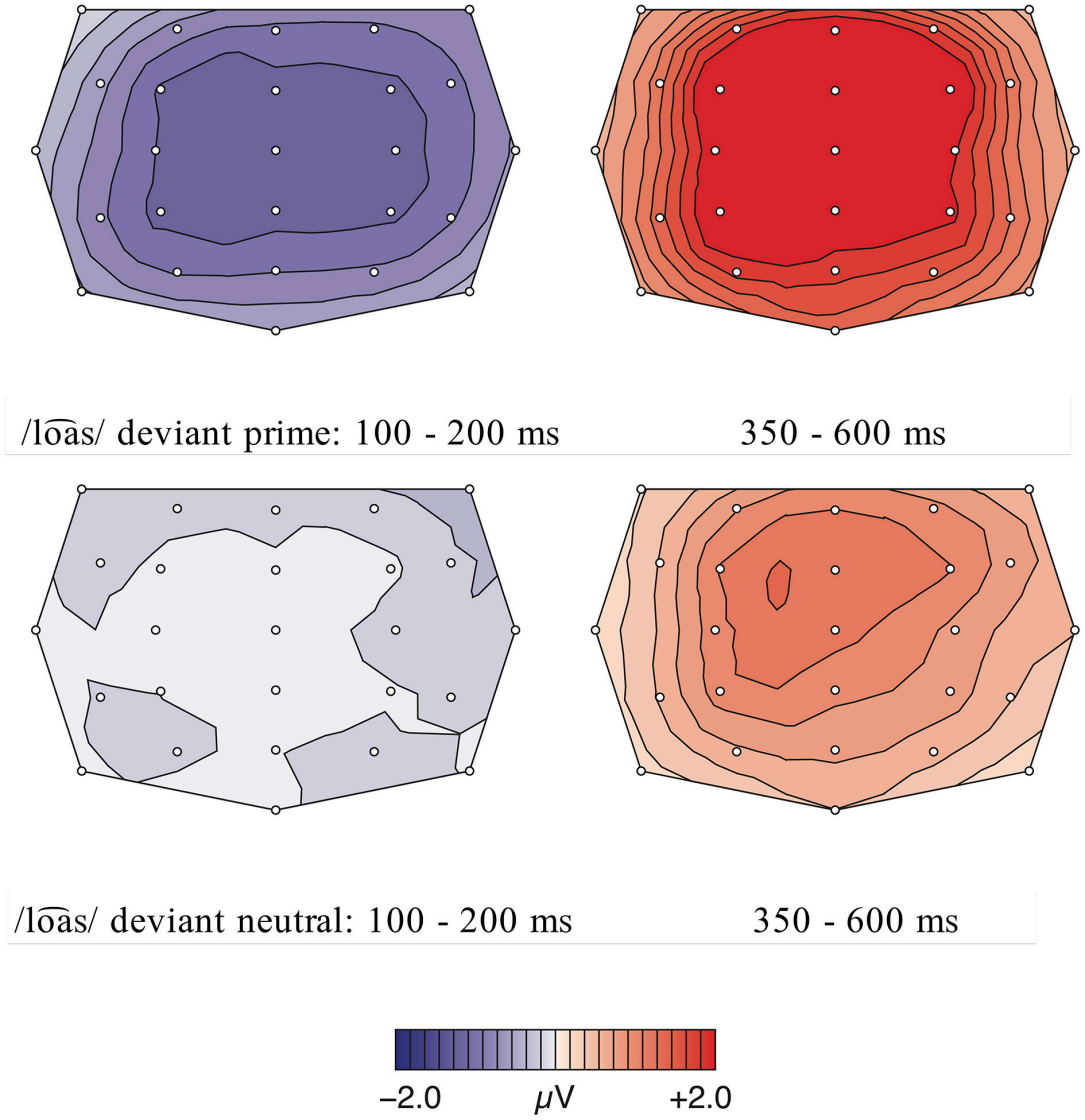
**(Top)** row: Condition 2, priming context: Topographic difference maps for the deviant condition /l$\overset{⌢}{\text{oa}}$s/ and control standard condition /l$\overset{⌢}{\text{o}Ʊ}$s/ for the significant time windows 100–200 ms and 350–600 ms. **(Lower)** row: Condition 2, neutral context: Topographic difference maps for the deviant condition /l$\overset{⌢}{\text{oa}}$s/ and control standard condition /l$\overset{⌢}{\text{o}Ʊ}$s/ for the time window 100–200 ms (n.s.) and the significant time window 350–600 ms.

The statistical analysis of the incomprehension condition calculating a repeated measures ANOVA with the factors word type (real word vs. pseudo-word) and region revealed an early negativity effect between 100 and 200 ms, elicited by the deviant pseudo-words (see Figures 5–7). This early negativity effect is only significant in the priming condition, leading to a significant main effect for the factor word type [*F*_(1, 17)_ = 28.88, *p* = 0.000, η^2^*p* = 0.08]. In contrast, there were no significant main effects in the neutral sentence context condition [word type: *F*_(1, 17)_ = 1.17, *p* > 0.05, η^2^*p* = 0.01; region: *F*_(2, 34)_ = 1.90, *p* > 0.05, η^2^*p* = 0.02]. In both contexts, no significant interaction between the two factors was observed [priming context: *F*_(2, 34)_ = 1.91, *p* > 0.05, η^2^*p* = 0.00 / neutral context: *F*_(2, 34)_ < 1, *p* > 0.05, η^2^*p* = 0.00]. In the priming context condition, this early negativity was followed by a late positive component (350–600 ms) [word type: *F*_(1, 17)_ = 23.33, *p* = 0.000, η^2^*p* = 0.15; region: *F*_(2, 34)_ = 19.66, *p* = 0.000, η^2^*p* = 0.09]. This late positivity was also significant in the neutral context condition [word type: *F*_(1, 17)_ = 22.96, *p* = 0.000, η^2^*p* = 0.08; region: *F*_(2, 34)_ = 29.14, *p* = 0.000, η^2^*p* = 0.15].

#### Discussion

The ERP results for the deviant l$\overset{⌢}{\text{oa}}$s_/l$\overset{⌢}{\text{o}Ʊ}$s_ in the priming condition show an early negative effect between 100 and 200 ms, which is absent in the neutral sentence context. We interpret this early negativity analogous to the negativity found for r$\overset{⌢}{\text{oa}}$s$\underset{ˈ}{\text{n}}$_/r$\overset{⌢}{\text{o}Ʊ}$s$\underset{ˈ}{\text{n}}$_ as the reflection of an early error detection mechanism. Due to the priming sentence context, the listener builds up strong expectancies toward the following incoming speech signal, i.e., for /l$\overset{⌢}{\text{o}Ʊ}$s/, which are violated when the deviating form /l$\overset{⌢}{\text{oa}}$s/ is perceived instead. Since only two different alternating phonological forms were presented, participants were able to differentiate the two lexemes very early, as /l$\overset{⌢}{\text{oa}}$s/ and /l$\overset{⌢}{\text{o}Ʊ}$s/ diverge from the onset of the diphthong in the F1 and F2 values (see Table 2), a fact reflected by the early latency of the negativity effect for l$\overset{⌢}{\text{oa}}$s_/l$\overset{⌢}{\text{o}Ʊ}$_. The absence of the N200 effect for neutral sentences emphasizes the influence of semantic priming on perception. Since the context does not provide any hints about the continuation of the sentence, there are no high expectancies built up and thus not violated.

Furthermore, an LPC (350–600 ms) was elicited for l$\overset{⌢}{\text{oa}}$s_/l$\overset{⌢}{\text{o}Ʊ}$s_ in the priming condition and in the neutral condition. We interpret the late positive deflection similarly as those elicited for condition 1 (see Section Discussion Condition 1: Misunderstanding). The amplitude of the LPC can also be modulated by the degree of complexity and difficulty, i.e., the resolvability of the given task: A more pronounced effect correlates with the simplicity of a stimulus according to the task requirement and thus with an easy evaluation (cf. Bentin et al., 1999; Domahs et al., 2009; Bohn et al., 2013). This relation is apparent in the ERP results for l$\overset{⌢}{\text{oa}}$s_/l$\overset{⌢}{\text{o}Ʊ}$s_, which show an enhanced positivity effect for primed sentences in contrast to neutral sentences. This indicates that the categorization of a stimulus is easier when participants are able to build up an expectation through priming. In neutral sentences, the context does not give indications about the sentences' progression, so participants cannot rely on clear cues for their evaluation. Thus, the rejection of the non-word /l$\overset{⌢}{\text{oa}}$s/ is more difficult for the participants, which is indicated by a reduced late positive component for l$\overset{⌢}{\text{oa}}$s_/l$\overset{⌢}{\text{o}Ʊ}$s_ in neutral sentences. Furthermore, the observed amplitude differences within this condition can also be explained by a reflected reanalysis process: in the priming context, the semantic cues enhance the expectations for a specific form; thus, the mismatch between the expected and encountered form is even bigger than in the neutral sentence context in which only the memory trace of the standard form built up an expectation. The stronger cues in the priming condition and thus the stronger degree of deviation are reflected in the more pronounced amplitude of the LPC in this sentence context condition.

### Condition 3: potential comprehension

#### Behavioral data

The analysis of the potential comprehension condition calculating an ANOVA with the factors phoneme and sentence context revealed only a significant main effect for the factor sentence context but no effect for the factor phoneme [sentence context: *F*_(1, 18)_ = 40.63, *p* = 0.000; phoneme: *F*_(1, 18)_ < 1, *p* > 0.05] and no significant interaction between the two factors [*F*_(1, 18)_ < 1, *p* > 0.05]. Thus, the acceptability and categorization process of the sentence-final word did not depend on the included phoneme but only on the sentence context the words were embedded in. Further paired comparisons of the arithmetical means showed that the standard form /lõː/ and the deviating form /l$\overset{⌢}{õ\tilde{Ʊ}}$/ were evaluated as equally acceptable sentence-final words in both sentence contexts [priming context: /lõː/, mean 1.31 (SD 0.45) vs. /l$\overset{⌢}{õ\tilde{Ʊ}}$/, mean 1.32 (SD 0.41); *Z*_(18)_ = −0.28, *p* > 0.05 / neutral context: /lõː/, mean 2.31 (SD 0.69) vs. /l$\overset{⌢}{õ\tilde{Ʊ}}$/, mean 2.33 (SD 0.60); *Z*_(18)_ = −0.24, *p* > 0.05]. However, the categorization was significantly easier in the priming context condition for both phoneme types [priming context: /lõː/, mean 1.31 (SD 0.45) vs. neutral context: /lõː/, mean 2.31 (SD 0.69); *Z*_(18)_ = −3.82, *p* = 0.000 / priming context: /l$\overset{⌢}{õ\tilde{Ʊ}}$/, mean 1.32 (SD 0.41) vs. neutral context: /l$\overset{⌢}{õ\tilde{Ʊ}}$/, mean 2.33 (SD 0.60); *Z*_(18)_ = −3.74, *p* = 0.000].

#### ERP data

**Figure 8:**
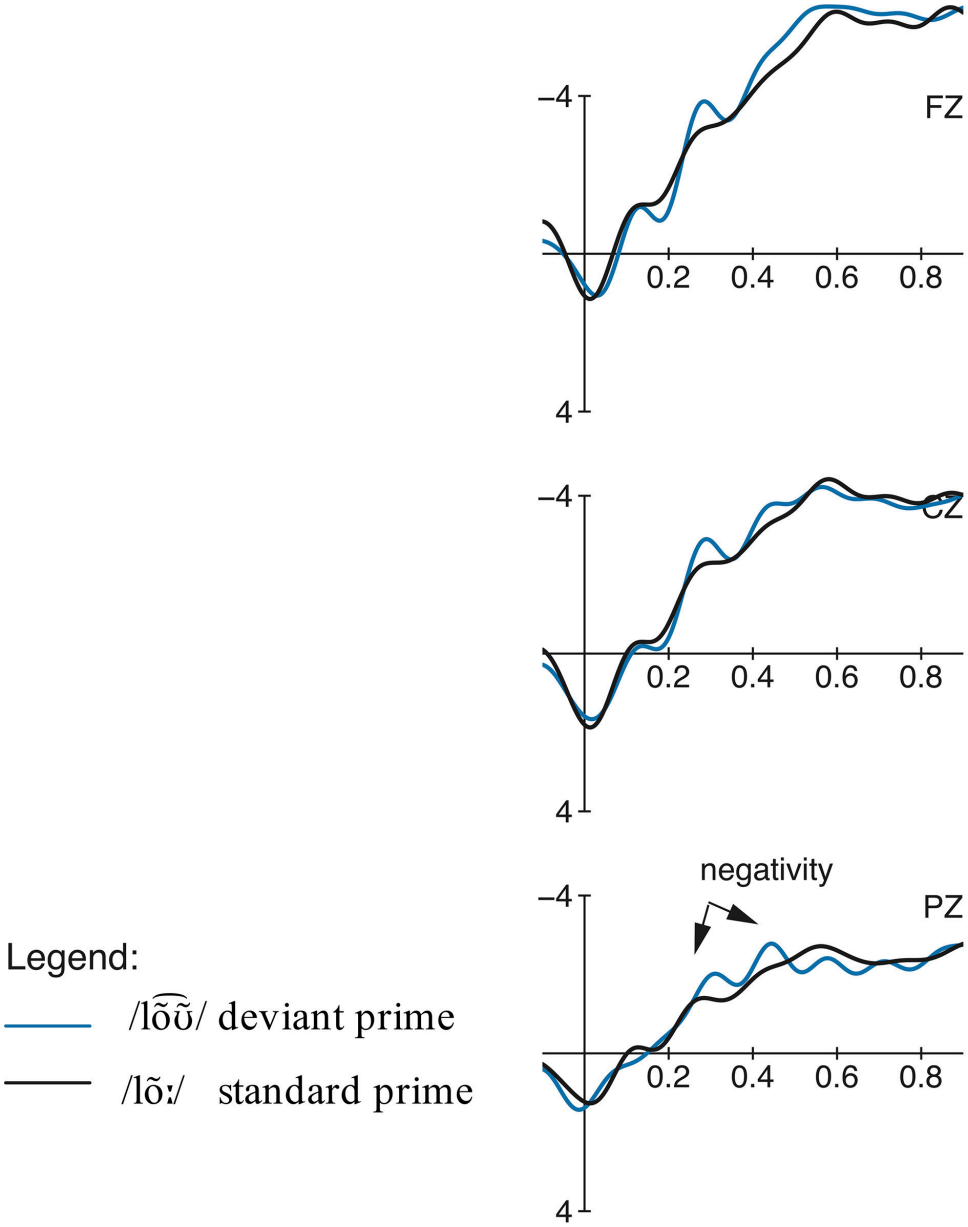
**Condition 3: Potential Comprehension, priming context: Grand averages of event-related potentials obtained for the deviant condition /l$\overset{⌢}{õ\tilde{Ʊ}}$/ and control standard condition /lõː/ measured from 100 ms prior the word onset up to 900 ms**.

**Figure 9:**
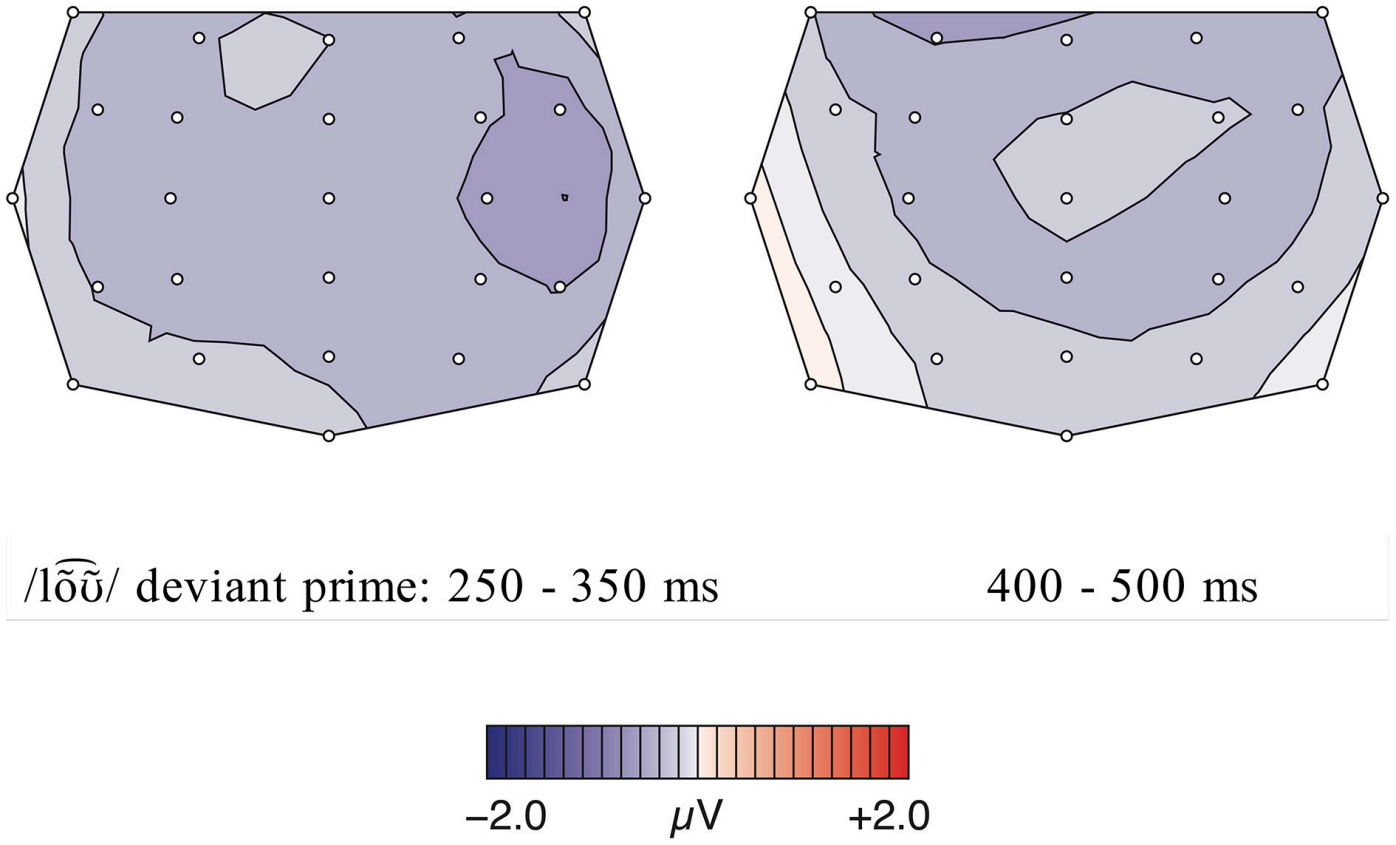
**Condition 3, priming context: Topographic difference maps for for the deviant condition /l$\overset{⌢}{õ\tilde{Ʊ}}$/ and control standard condition /lõː/ for the significant time windows 250–350 ms and 400–500 ms**.

**Figure 10:**
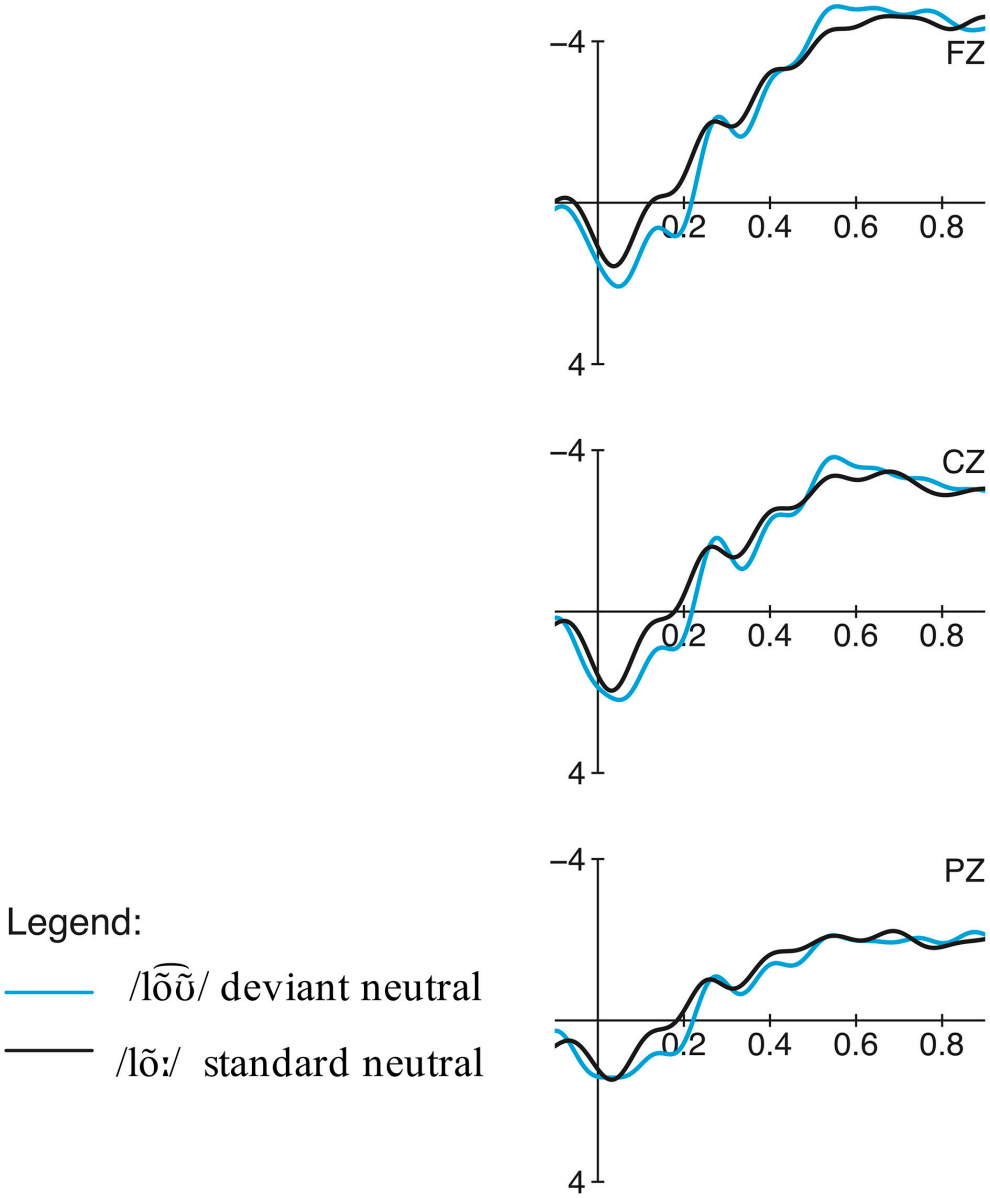
**Condition 3: Potential Comprehension, neutral context: Grand averages of event-related potentials obtained for the deviant condition /l$\overset{⌢}{õ\tilde{Ʊ}}$/ and control standard condition /lõː/ measured from 100 ms prior the word onset up to 900 ms**.

The comparison of the standard form and the deviant form in the potential comprehension condition revealed two moderate consecutive negativity effects, elicited by the deviant condition in the time windows 250–350 ms and 400–500 ms (see Figures 8–10). The repeated measures ANOVA showed significant main effects for the factors phoneme (standard /lõː/ vs. deviant /l$\overset{⌢}{õ\tilde{Ʊ}}$/) and region in the first time window [phoneme: *F*_(1, 18)_ = 5.26, *p* = 0.034, η^2^*p* = 0.01; region: *F*_(2, 36)_ = 14.67, *p* = 0.001, η^2^*p* = 0.11] as well as in the second time window [phoneme: *F*_(1, 18)_ = 7.46, *p* = 0.014, η^2^*p* = 0.01; region: *F*_(2, 36)_ = 15.93, *p* = 0.001, η^2^*p* = 0.15] but no significant interactions between these two factors. The same comparison embedded in neutral sentence context did not reveal any significant effects in any time window.

#### Discussion

With regard to the ERP results, two moderate negativities for l$\overset{⌢}{õ\tilde{Ʊ}}$_∕lõː_ between 250–350 ms and 400–500 ms were obtained, which are absent in the neutral condition. In contrast to the other conditions, the amplitude of these negative deflections is much less pronounced. Although /l$\overset{⌢}{\text{oa}}$s/ as well as /l$\overset{⌢}{õ\tilde{Ʊ}}$/ both do not match the native Central Bavarian lexemes, they differ in their phonetic distance to the native variants. In contrast to pseudo-words, the lexemes /lõː/ and /l$\overset{⌢}{õ\tilde{Ʊ}}$/ are related and thus do not elicit the same pronounced negativity as unrelated words do (cf. Kutas and Van Petten, 1994). Thus, the reduced negative deflections in contrast to the more pronounced one for l$\overset{⌢}{\text{oa}}$s_/l$\overset{⌢}{\text{o}Ʊ}$s_ can be interpreted as simply reflecting the perception of a phonetic deviation and thus a slightly hindered lexical access caused by the phonetically deviating form. This is in contrast to results from an MMN study on Italian vowel contrasts by Miglietta et al. (2013) who found similarly pronounced effects for both an allophonic ([ε]-[e]) and phonemic ([e]-[i]) condition. However, since this study concentrated on the processing of isolated vowels without lexical or semantic context, this does not refute our findings. This is in line with the fact that the sentence context plays a decisive role, since the negativities are only elicited when the meaning ‘wage’ has already been pre-activated through priming. In neutral contexts, however, no negative effects could be observed since no strong expectations toward a special form had been built up in these sentences.

Furthermore, the behavioral data show no difference in the rating between the match and mismatch condition, indicating that the deviant /l$\overset{⌢}{õ\tilde{Ʊ}}$/ is also interpreted in terms of the meaning ‘wage’. However, the evaluation process was easier in the priming context, i.e., the cues of semantic expectation facilitated the categorization process for both forms in these sentences.

Table 3 gives an overview of the most important results of the behavioral data. Table 4 displays an overview of relevant time windows and significant ERP results for all conducted comparisons of the three conditions described in the sections ERP data Condition 1: Misunderstanding– ERP data Condition 3: Potential comprehension. Table 5 contains mean latency and amplitude values measured from the difference waves of all statistically significant comparisons.

**Table 3:** **Behavioral data: mean evaluations of all responses for each standard-deviant pair of each condition (scale from 1 = acceptable to 4 = unacceptable)**.

**Condition**	**Sentence context**	**Evaluation (mean)**	***p*-value**	**Critical items**
Misunderstanding	Priming /r$\overset{⌢}{\text{oa}}$s$\underset{ˈ}{\text{n}}$/	standard: 1.15	0.000^***^	/r$\overset{⌢}{\text{oa}}$s$\underset{ˈ}{\text{n}}$/ ‘journeys’ vs. /r$\overset{⌢}{\text{o}Ʊ}$s$\underset{ˈ}{\text{n}}$/ ‘roses’
		deviant: 3.90		
	Priming /r$\overset{⌢}{\text{o}Ʊ}$s$\underset{ˈ}{\text{n}}$/	standard: 1.12	0.000^***^	/r$\overset{⌢}{\text{o}Ʊ}$s$\underset{ˈ}{\text{n}}$/ ‘roses’ vs. /r$\overset{⌢}{\text{oa}}$s$\underset{ˈ}{\text{n}}$/ ‘journeys’
		deviant: 3.72		
Incomprehension	Priming /l$\overset{⌢}{\text{o}Ʊ}$s/	standard: 1.74	>0.05 n.s.	/l$\overset{⌢}{\text{o}Ʊ}$s/ ‘sow’ vs. /l$\overset{⌢}{\text{oa}}$s/ pseudo-word
		deviant: 2.72		
	Neutral	standard: 2.41	0.020^*^	
		deviant: 3.07		
Potential comprehension	Priming /lõː/	standard: 1.31	>0.05 n.s.	/lõː/ ‘wage’ vs. /l$\overset{⌢}{\text{o}Ʊ}$/ potential allophonic form
		deviant: 1.32		
	Neutral	standard: 2.31	>0.05 n.s.	
		deviant: 2.33		

Statistical significance is indicated by

* p < 0.05, ^**^p < 0.01,

*** *p < 0.001*.

**Table 4:** **ERP effects in different time windows for three different conditions**.

**Condition**	**Sentence context**	**Negativity**	**Positivity**	**Critical items**
Misunderstanding	Priming /r$\overset{⌢}{\text{oa}}$s$\underset{ˈ}{\text{n}}$/	100–200^**^	400–900^***^	/r$\overset{⌢}{\text{o}Ʊ}$s$\underset{ˈ}{\text{n}}$/ ‘roses’ vs. /r$\overset{⌢}{\text{oa}}$s$\underset{ˈ}{\text{n}}$/ ‘journeys’
	Priming /r$\overset{⌢}{\text{o}Ʊ}$s$\underset{ˈ}{\text{n}}$/	300–500^**^	550–1000^***^	
Incomprehension	Priming /l$\overset{⌢}{\text{o}Ʊ}$s/	100–200^***^	350–600^***^	/l$\overset{⌢}{\text{o}Ʊ}$s/ ‘sow’ vs. /l$\overset{⌢}{\text{oa}}$s/ pseudo-word
	Neutral	100–200 n.s.	350–600^***^	
Potential comprehension	Priming /lõː/	250–350^*^	–	/lõː/ ‘wage’ vs. /l$\overset{⌢}{\text{o}Ʊ}$/ potential allophonic form
		400–500^*^		
	Neutral	–	–	

Statistical significance is indicated by

* *p < 0.05*,

** *p < 0.01*,

*** *p < 0.001*.

**Table 5:** **Peak latencies and amplitudes of significant ERP components measured over nine electrodes (F3, FZ, F4, C3, CZ, C4, P3, PZ, P4), standard deviations given in brackets**.

**Condition**	**Comparison**	**Mean peak latency in ms (SD)**	**Mean peak amplitude in μV (SD)**	**Critical items**
Misunderstanding	r$\overset{⌢}{\text{oa}}$s$\underset{ˈ}{\text{n}}$_/$\overset{⌢}{\text{o}Ʊ}$s$\underset{ˈ}{\text{n}}$_	Neg: 132 (6.82)	Neg: −0.88 (0.12)	/r$\overset{⌢}{\text{oa}}$s$\underset{ˈ}{\text{n}}$/ ‘journeys’ vs. /r$\overset{⌢}{\text{o}Ʊ}$s$\underset{ˈ}{\text{n}}$/ ‘roses’
		Pos: 517 (91.21)	Pos: 2.94 (0.53)	
	r$\overset{⌢}{\text{o}Ʊ}$s$\underset{ˈ}{\text{n}}$_/r$\overset{⌢}{\text{oa}}$s$\underset{ˈ}{\text{n}}$_	Neg: 400 (14.58)	Neg: −1.77 (0.44)	/r$\overset{⌢}{\text{o}Ʊ}$s$\underset{ˈ}{\text{n}}$/ ‘roses’ vs. /r$\overset{⌢}{\text{oa}}$s$\underset{ˈ}{\text{n}}$/ ‘journeys’
		Pos: 664 (82.96)	Pos:2.14 (0.40)	
Incomprehension	Priming context l$\overset{⌢}{\text{oa}}$s_/l$\overset{⌢}{\text{o}Ʊ}$_	Neg: 154 (3.71)	Neg: −1.43 (0.19)	/l$\overset{⌢}{\text{o}Ʊ}$s/ ‘sow’ vs. /l$\overset{⌢}{\text{oa}}$s/ pseudo-word
		Pos: 500 (3.71)	Pos: 3.41 (0.39)	
	Neutral context l$\overset{⌢}{\text{oa}}$s_/l$\overset{⌢}{\text{o}Ʊ}$_	Pos: 485 (18.92)	Pos: 1.68 (0.24)	
Potential comprehension	Priming context l$\overset{⌢}{õ\tilde{Ʊ}}$_/lõː_	Neg: 292 (15.46)	Neg: −0.74 (0.15)	/lõː/ ‘wage’ vs. /l$\overset{⌢}{õ\tilde{Ʊ}}$/ potential allophonic form
		Neg: 450 (34.23)	Neg: −0.61 (0.11)	
	Neutral context l$\overset{⌢}{õ\tilde{Ʊ}}$_∕lõː_	—	—	

## General discussion

The present paper explores the potential difficulties in cross-dialectal comprehension attributable to the close contact of a merged and an unmerged speech community. In contrast to previous studies, the investigated phonemes /$\overset{⌢}{\text{oa}}$/ and /$\overset{⌢}{\text{o}Ʊ}$/ occur in both dialect areas, but are assigned to different lexemes. In fact, we found different ERP effects for three different types of potential communication difficulties. These are discussed first with regard to their asymmetric latency distribution found within the misunderstanding condition and second in terms of the connection between cross-dialectal comprehension and phoneme change.

### Asymmetric ERP effects in misunderstanding condition

In the misunderstanding condition, we expected to find an N400 effect for both deviant types due to the identical semantic priming context. However, while we indeed found a centro-parietal negativity between 300 and 500 ms for r$\overset{⌢}{\text{o}Ʊ}$s$\underset{ˈ}{\text{n}}$_/r$\overset{⌢}{\text{oa}}$s$\underset{ˈ}{\text{n}}$_, the negativity for r$\overset{⌢}{\text{oa}}$s$\underset{ˈ}{\text{n}}$_/r$\overset{⌢}{\text{o}Ʊ}$s$\underset{ˈ}{\text{n}}$_ was elicited in an earlier time window between 100 and 200 ms. This is somewhat unexpected, since the same phonetic material for the critical items was used and behavioral data show a significant difference between both predictable and unpredictable sentence-final lexemes, suggesting that the forms were perceived differently and were easy to distinguish. One reason for the absence of an N400 effect might be the low variability of semantically alternating sentence-final word forms, as Bendixen et al. (2014) suggest. Therefore, it is possible that both words were semantically activated during the experiment. Furthermore, studies have shown that a high degree of repetition is responsible for abolishment or decrease of the N400 amplitude (cf. Conrey et al., 2005; Renoult et al., 2012). However, since the deviant r$\overset{⌢}{\text{o}Ʊ}$s$\underset{ˈ}{\text{n}}$_/r$\overset{⌢}{\text{oa}}$s$\underset{ˈ}{\text{n}}$_ elicited a centro-parietal negativity effect in the typical latency range of the N400 component, these explanations can be regarded as rather unlikely.

Another possible reason for the absence of an N400 for this particular deviant form is its frequency. It is likely that the dialectal lexeme for ‘journeys’ has a lower frequency than the one for ‘roses’. In fact, the modulation of the N400 is known to be sensitive to word frequency (cf. Kutas and Federmeier, 2000, 2011; Lau et al., 2008). Unfortunately, no data are available for word frequencies in dialects, which may differ strongly from the standard variety so that this interpretation can neither be supported nor weakened by word frequency data.

The latency differences might also be due to the diphthong itself, since studies show that early negative components are modulated by specific features of the respective phonemes. In the featurally underspecified lexicon model (FUL) by Lahiri and Reetz (2002, 2010), asymmetries in vowel perception are explained by underlying phonological features. Eulitz and Lahiri (2004) argue that for an underspecified deviant preceded by a specified standard, the MMN reflects an acoustic and feature mismatch, while no mismatch is given for the reverse direction (see also Scharinger et al., 2012). This model cannot be adapted to the /$\overset{⌢}{\text{oa}}$/-/$\overset{⌢}{\text{o}Ʊ}$/ contrast one-to-one, but underspecification can be understood as an overall representation of a speech sound, as well. Thus, articulatory more specific speech sounds are reflected by specific tongue positions, which require more detailed motor planning (cf. Scharinger et al., 2012). Applied to the diphthongs in our study, /$\overset{⌢}{\text{oa}}$/ and /$\overset{⌢}{\text{o}Ʊ}$/ differ in the articulatory effort of their respective tongue movement, indicated by the F1 and F2 values. The difference between the formant values between 20% and 80% of the diphthong is −127.1 (F1) and −471.7 (F2) for /$\overset{⌢}{\text{oa}}$/, respectively 122.1 (F1) and 89.9 (F2) for /$\overset{⌢}{\text{o}Ʊ}$/, indicating that the tongue movement in the diphthong /$\overset{⌢}{\text{o}Ʊ}$/ is less pronounced than in /$\overset{⌢}{\text{oa}}$/. In this regard, it can be assumed that /$\overset{⌢}{\text{oa}}$/ requires more specific motor planning than /$\overset{⌢}{\text{o}Ʊ}$/, so that the asymmetric effects found for r$\overset{⌢}{\text{oa}}$s$\underset{ˈ}{\text{n}}$_/r$\overset{⌢}{\text{o}Ʊ}$s$\underset{ˈ}{\text{n}}$_ and r$\overset{⌢}{\text{o}Ʊ}$s$\underset{ˈ}{\text{n}}$_/r$\overset{⌢}{\text{oa}}$s$\underset{ˈ}{\text{n}}$_ may also reflect the different articulatory effort.

**Figure 11:**
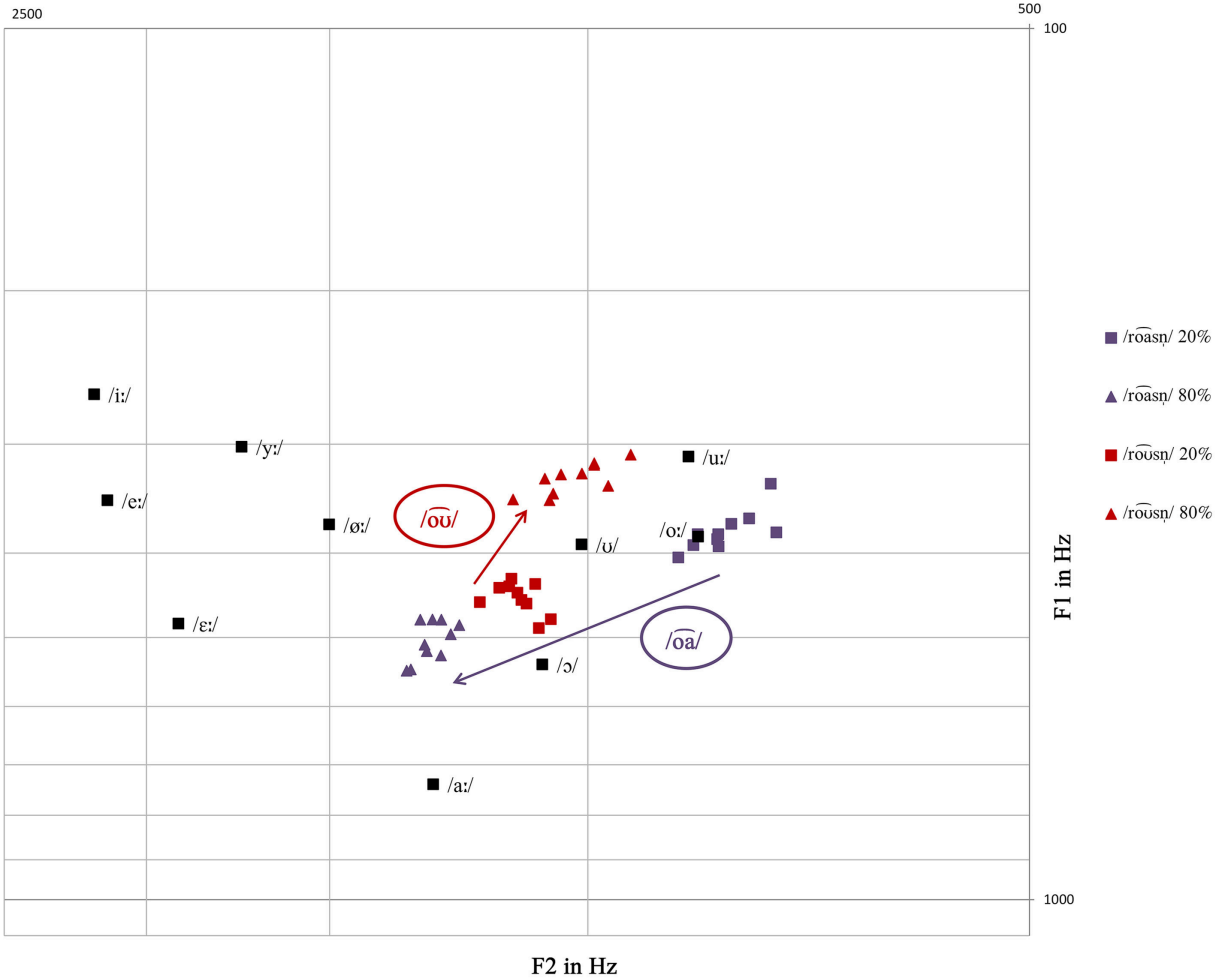
**Measured formant values of /$\overset{⌢}{\text{oa}}$/ and /$\overset{⌢}{\text{o}Ʊ}$/ of the 10 /r$\overset{⌢}{\text{oa}}$s$\underset{ˈ}{\text{n}}$/-/r$\overset{⌢}{\text{o}Ʊ}$s$\underset{ˈ}{\text{n}}$/ items mapped on the Standard German vowel system measured by Sendlmeier and Seebode (2007)**.

The fact that the lexical-semantic processing of the two lexemes is heavily influenced by the two different diphthongs is further supported by data on asymmetric vowel perception from Polka and Bohn (2003). For infants, the discrimination of a vowel change presented in the direction from a less peripheral to a more peripheral vowel, i.e., closer to the limits or corners of the vowel space, is significantly better than in the reversed direction (see also Polka and Werker, 1994; Polka and Bohn, 1996). The authors conclude that the relatively more peripheral vowel in a contrast serves as a reference point. This is related to the assumption that asymmetries in vowel perception result from the salience of vowels, which is innately more pronounced for vowels tied to extremes of the vowel space. The peripheral hypothesis can be confirmed on the lexical level as well (Sebastián-Gallés et al., 2005; Larsson et al., 2008), indicating that such perceptual asymmetries reflect higher-level processing at phonetic and lexical levels rather than a low-level general auditory processing (Polka and Bohn, 2011). This assumption fits well with the results obtained in our study examining full lexical words. The formant map of /$\overset{⌢}{\text{oa}}$/ and /$\overset{⌢}{\text{o}Ʊ}$/ shows that at the 20% measurement point, /$\overset{⌢}{\text{oa}}$/ is the more peripheral vowel in this contrast (see Figure 11). Assuming the peripheral hypothesis, the deviant r$\overset{⌢}{\text{oa}}$s$\underset{ˈ}{\text{n}}$_/r$\overset{⌢}{\text{o}Ʊ}$s$\underset{ˈ}{\text{n}}$_ is suggested to be discriminated more easily than the deviant r$\overset{⌢}{\text{o}Ʊ}$s$\underset{ˈ}{\text{n}}$_/r$\overset{⌢}{\text{oa}}$s$\underset{ˈ}{\text{n}}$_, since in the former condition the direction of change is from the less peripheral vowel /$\overset{⌢}{\text{o}Ʊ}$/ to the more peripheral /$\overset{⌢}{\text{oa}}$/. This easier discriminability is reflected by the earlier negativity effect for r$\overset{⌢}{\text{oa}}$s$\underset{ˈ}{\text{n}}$_/r$\overset{⌢}{\text{o}Ʊ}$s$\underset{ˈ}{\text{n}}$_ in contrast to r$\overset{⌢}{\text{o}Ʊ}$s$\underset{ˈ}{\text{n}}$_/r$\overset{⌢}{\text{oa}}$s$\underset{ˈ}{\text{n}}$_, indicating a facilitated discrimination of the vowel /$\overset{⌢}{\text{oa}}$/ due to its more peripheral position in the vowel space and thus its salience. The higher saliency of the /$\overset{⌢}{\text{oa}}$/-diphthong might also be due to the fact that it is a falling diphthong which is a rather uncommon phenomenon in German varieties. Since asymmetries in vowel perception can be observed even in native listeners when lexical processing is involved in the task (cf. Polka and Bohn, 2011), the peripheral position of the /$\overset{⌢}{\text{oa}}$/-diphthong might be an influencing factor concerning the relative earliness of the negativity effect for r$\overset{⌢}{\text{oa}}$s$\underset{ˈ}{\text{n}}$_/r$\overset{⌢}{\text{o}Ʊ}$s$\underset{ˈ}{\text{n}}$_ in contrast to r$\overset{⌢}{\text{o}Ʊ}$s$\underset{ˈ}{\text{n}}$_/r$\overset{⌢}{\text{oa}}$s$\underset{ˈ}{\text{n}}$_. Although the negativities found display different latencies, it is important to keep in mind that both lexemes elicited an error detection mechanism induced by the semantic priming, leading to a problematic lexical retrieval for both forms.

### Cross-dialectal comprehension and phoneme change

Data from dialectology studies show that a change of the /$\overset{⌢}{\text{oa}}$/-phoneme can be detected in several lexemes in BA which is thought to be due to communicative interactions between speakers of BA and CB who synchronize their different linguistic competencies. Synchronization in our view refers to the calibration of competence differences in the performance act, which results in a stabilization or modification of the active and passive competencies involved (cf. Schmidt, 2010; Schmidt and Herrgen, 2011). Desiring to be understood, listeners actively and interactively synchronize their individual competencies. In every speech production, speakers try to match their individual competences to their interlocutor's communicative expectations and abilities in order to be understood. Negative feedback from the interlocutor signaling a lack of comprehension or only partial comprehension leads to modification of the applied language production strategy effecting a reconstruction of the speaker's individual competence (so-called microsynchronization; cf. Schmidt, 2010; Schmidt and Herrgen, 2011). In long-term contact situations (e.g., school classes, youth peer groups, regional groups), subjects interact over an extended period of time. This leads to a series of parallel synchronizations implying repetitive positive or negative feedback. The result is a congruent part of linguistic knowledge, which enables the communication partners to communicate successfully. Such a series of parallel acts of synchronization performed by individuals in personal contact situations, which lead to the establishment of common context-dependent linguistic knowledge, are designated as so-called mesosynchronizations (cf. Schmidt, 2010; Schmidt and Herrgen, 2011)^2^.

The lexically gradual change of the /$\overset{⌢}{\text{oa}}$/-diphthong in the Bavarian-Alemannic transition zone may be due to such mesosynchronizations. In this regionally defined speaker group, a constant stream of parallel synchronizations led to parallel optimization strategies resulting in a convergence to the dominant Central Bavarian variant. Thus, the synchronizations led to a gradual modification of the group's linguistic competence, which is reflected by a word-by-word phonological redistribution of the /$\overset{⌢}{\text{oa}}$/-diphthong. This gradual change can also be explained by the interactional approach. During the adaptation process, speakers modify their pronunciation of particular words rather than the complete phonological system at once. Some words are thus affected earlier by the change than others. This is caused by the speakers' motivation to pronounce individual lexemes just as speakers from the target variety (cf. Trudgill, 1986).

The current study focuses on the question whether this slow but continuous change in the usage of the dialectal /$\overset{⌢}{\text{oa}}$/-phoneme leads to increased neural costs during sentence processing. In fact, the results display clear processing differences between the deviants r$\overset{⌢}{\text{oa}}$s$\underset{ˈ}{\text{n}}$_/r$\overset{⌢}{\text{o}Ʊ}$s$\underset{ˈ}{\text{n}}$_ and l$\overset{⌢}{\text{oa}}$s_/l$\overset{⌢}{\text{o}Ʊ}$s_ in contrast to l$\overset{⌢}{õ\tilde{Ʊ}}$_∕lõː_. The aforementioned forms elicit early negativities (N2b) associated with an early error detection mechanism, which is absent for /l$\overset{⌢}{õ\tilde{Ʊ}}$/. Furthermore, r$\overset{⌢}{\text{oa}}$s$\underset{ˈ}{\text{n}}$_/r$\overset{⌢}{\text{o}Ʊ}$s$\underset{ˈ}{\text{n}}$_ and l$\overset{⌢}{\text{oa}}$s_/l$\overset{⌢}{\text{o}Ʊ}$s_ elicit LPCs reflecting an evaluation process of the previously detected mismatch, which is also absent for /l$\overset{⌢}{õ\tilde{Ʊ}}$/.

The effects found for /r$\overset{⌢}{\text{oa}}$s$\underset{ˈ}{\text{n}}$/ and /l$\overset{⌢}{\text{oa}}$s/ can be interpreted within the scope of the synchronization theory, as the detection and evaluation of erroneous usage of the native form /r$\overset{⌢}{\text{oa}}$s$\underset{ˈ}{\text{n}}$/ and the unknown form /l$\overset{⌢}{\text{oa}}$s/ lead to negative feedback. Our data suggest that both misunderstanding as well as incomprehension induce similar neural costs during cross-dialectal comprehension. In both cases, the non-native variant leads to increased costs at an early stage of processing reflecting the detection of an error in the sentential context. Thus, these cross-dialectal competence differences do lead to cross-dialectal comprehension difficulties, which are reflected in both neural responses as well as in behavioral data. These difficulties can be interpreted as a trigger for phoneme change, since the error detection could be proven for r$\overset{⌢}{\text{oa}}$s$\underset{ˈ}{\text{n}}$_/r$\overset{⌢}{\text{o}Ʊ}$s$\underset{ˈ}{\text{n}}$_—a lexeme involved in language change in BA, amongst others.

However, the conditions misunderstanding and incomprehension differ partially with regard to the early negativity's amplitude. Surprisingly, the amplitude of the N2b is less pronounced for r$\overset{⌢}{\text{oa}}$s$\underset{ˈ}{\text{n}}$_/r$\overset{⌢}{\text{o}Ʊ}$s$\underset{ˈ}{\text{n}}$_, although the behavioral data show a significant effect between standard and deviant. Moreover, the behavioral data show that, although there is a significant difference in the evaluation of deviant and standard, the unacceptability rates for the reversed misunderstanding contrast are higher. It thus seems as if the deviant form /r$\overset{⌢}{\text{oa}}$s$\underset{ˈ}{\text{n}}$/ was not as easily rejectable as its counterpart /r$\overset{⌢}{\text{o}Ʊ}$s$\underset{ˈ}{\text{n}}$/. This might be attributed to the close contact situation of the participants to the merged community, as the exposure to regional variability influences the perception and processing of vowel contrasts in their own accent as well. Hence, although the respective vowel contrast is still preserved in CB, the less clear rejection of r$\overset{⌢}{\text{oa}}$s$\underset{ˈ}{\text{n}}$_/r$\overset{⌢}{\text{o}Ʊ}$s$\underset{ˈ}{\text{n}}$_ indicates a level of uncertainty during lexical and evaluation processing caused by the exposure to regional variability. These results are in line with Brunellière et al. (2009) who show that listeners who preserve a particular phonological contrast, but are often exposed to merged variants, discriminate this contrast less easily than regional stable contrasts. Conrey et al. (2005) could further show that unmerged dialect speakers are still worse at distinguishing between merged vowels than other vowels unaffected by a merger.

This influence of the close dialect contact is also visible in the results for the incomprehension condition. As a deviant, the well-formed pseudo-word /l$\overset{⌢}{\text{oa}}$s/, which is non-existent in the listeners' lexicon, was presented. However, /l$\overset{⌢}{\text{oa}}$s/ is indeed an existing word in the adjacent dialect of BA. This might be a further reason for the fact that we did not find an N400 effect, which is normally elicited by unknown novel word forms, as they require additional processing resources in order to classify them as non-existing words (cf. Bentin et al., 1999; Domahs et al., 2009). Further evidence comes from the behavioral data: Although the pseudo-word is less acceptable in comparison to the real word form /l$\overset{⌢}{\text{o}Ʊ}$s/, no significant effect between the match and mismatch condition could be found for primed sentences. The wide dispersion of the standard deviation in this condition suggests uncertainties when evaluating the sentences, indicating a more extensive evaluation of its lexical status and thus semantic classification. This process could be impeded by the circumstance that the pseudo-word is the correct phonemic form for the same semantic concept in the neighboring dialect area. Thus, especially in the priming context, the classification of the deviating form is especially problematic for the participants. This fact is also reflected in the high dispersion of the standard deviation (cf. Section ERP Data in Condition 1: Misunderstanding).

Unlike the conditions 1 and 2, neither ERP nor behavioral data show comparable results for the potential comprehension condition. Although the deviant /l$\overset{⌢}{õ\tilde{Ʊ}}$/ is also a non-existing pseudo-word, it is phonetically very similar to the native lexeme. The absence of an LPC and the similar acceptability rates for both forms indicate that no reanalysis-mechanism was required and that the evaluation of both forms was equally easy to categorize. This suggests that both forms are associated with the meaning ‘wage’, i.e., the phonetically deviant variant /l$\overset{⌢}{õ\tilde{Ʊ}}$/ is perceived as an allophonic variant of /lõː/, even though it is not an appropriate form in the listener's competence. It does thus not elicit a mismatch between expectation and perceived input, as both forms were equally categorized as existing word forms. Henrich et al. (2015) support the unproblematic evaluation of both critical and control conditions illustrated by a similar absence of a P300 in the evaluation process of rhythmically well-formed and subtle deviant structures. The fact that allophonic variability does not lead to enhanced processing costs, while phonological contrasts (r$\overset{⌢}{\text{oa}}$s$\underset{ˈ}{\text{n}}$_/r$\overset{⌢}{\text{o}Ʊ}$s$\underset{ˈ}{\text{n}}$_ and l$\overset{⌢}{\text{oa}}$s_/l$\overset{⌢}{\text{o}Ʊ}$s_) elicit clear ERP effects is in line with results from Kazanina et al. (2006), who found an MMNm for the phonemic [t]-[d] contrast for Russian listeners, which is absent for Koreans for whom the consonants are purely allophonic.

Thus, the usage of the /$\overset{⌢}{\text{o}Ʊ}$/-diphthong in lexemes going back to MHG *ô* does not lead to comprehension difficulties and the change from /$\overset{⌢}{\text{oa}}$/ to /$\overset{⌢}{\text{o}Ʊ}$/ seems to be a worthwhile strategy to reduce both misunderstandings/incomprehension and neural costs in processing during cross-dialectal communication.

## Conclusion

The aim of the present ERP study was to examine the speech processing of Central Bavarian speakers, who are exposed to certain lexemes common to the Bavarian-Alemannic transition zone. The speaker groups differ with regard to their linguistic competence insofar as a phonemic merger of MHG *ô* and MHG *ei* to /$\overset{⌢}{\text{oa}}$/ took place in the Bavarian-Alemannic transition zone. By contrast, in the Central Bavarian dialect two phonemes are distinguished (/$\overset{⌢}{\text{oa}}$/-/$\overset{⌢}{\text{o}Ʊ}$/). It is assumed that these competence differences lead to communication problems between the dialect areas.

To approach this particular cross-dialectal communication setting, a more natural design in which listeners are exposed to complex sentences was created in combining an oddball design with whole sentences and a semantic rating task. This advanced and innovative design is quite promising, as single minimal pairs can be investigated during sentence processing.

Results show robust effects despite the low variance of critical items and recurrent sentence contexts. Indeed, the Bavarian-Alemannic lexemes /l$\overset{⌢}{\text{oa}}$s/ ‘sow’ and /r$\overset{⌢}{\text{oa}}$s$\underset{ˈ}{\text{n}}$/ ‘roses’ evoke enhanced neural costs during sentence comprehension, reflected by biphasic ERP patterns consisting of an N200 and an LPC. These results demonstrate a conscious mismatch detection and evaluation process with regard to the non-native lexemes. Thus, it seems likely that the usage of these lexemes cause problems during cross-dialectal communication, leading to negative feedback and subsequently to competence modifications. In contrast, the results for the lexeme /l$\overset{⌢}{õ\tilde{Ʊ}}$/ show only reduced negativity effects and no LPC suggesting that in this case allophonic deviations are perceived but do not prevent successful communication.

To conclude, the results reveal first ERP evidence for cross-dialectal misunderstanding when speakers from the merged area use the /$\overset{⌢}{\text{oa}}$/-diphthong stemming from MHG *ô*. No similar effects could be detected for the phonetically related /$\overset{⌢}{\text{o}Ʊ}$/-phoneme. The empirically observable change of /$\overset{⌢}{\text{oa}}$/ to either /$\overset{⌢}{\text{o}Ʊ}$/ or /oː/ in the Bavarian-Alemannic transition zone can thus be interpreted as a strategy to avoid costly communication difficulties in close dialect contact settings. Insofar, dialectal competence differences resulting in enhanced neural processing costs can indeed trigger dialect change in order to facilitate successful cross-dialectal communication.

## Author contributions

ML conception of experimental stimuli and experimental paradigm; phonetic analysis of auditory stimuli; data acquisition; interpretation of results; preparation of the manuscript's first draft, revision of the manuscript. KH conception of experimental stimuli and experimental paradigm; data acquisition; data analysis; interpretation of results; preparation of the manuscript's first draft, revision of the manuscript. MR conception of experimental stimuli and experimental paradigm; phonetic analysis of auditory stimuli; data acquisition; interpretation of results; proofreading. HS conception of experimental stimuli and experimental paradigm; phonetic analysis of auditory stimuli; data acquisition; interpretation of results. AW conception of experimental stimuli and experimental paradigm; phonetic analysis of auditory stimuli; data acquisition; interpretation of results; proofreading. JH conception of experimental paradigm; interpretation of results; proofreading. JS conception of experimental paradigm; interpretation of results; proofreading.

## Funding

The research presented here is supported by the Hessen State Ministry of Higher Education, Research and the Arts, Landesoffensive zur Entwicklung wissenschaftlich-ökonomischer Excellenz (LOEWE), Research Focus: Exploring fundamental linguistic categories grant, research project Phonological word–Constituents of the Phonological Word under the leadership of JH, JS, and AW.

### Conflict of interest statement

The authors declare that the research was conducted in the absence of any commercial or financial relationships that could be construed as a potential conflict of interest.

## Footnotes

^1^Dialects can be defined as the least standard and most local (regionally restricted) varieties of a language and consist of independent prosodic, phonological and morpho-syntactic structures (cf. Schmidt, 2011).

^2^The accommodation theory developed by Howard Giles focuses on questions about the convergence in face-to-face interaction, as well. It is not possible to discuss theoretical differences between the theories of accommodation and synchronization within the scope of this paper.
