# Supplementary material for: Dialect Variation Influences the Phonological and Lexical-Semantic Word Processing in Sentences. Electrophysiological Evidence from a Cross-Dialectal Comprehension Study
Source: Front Psychol. 2016 May 27;7:739. doi: 10.3389/fpsyg.2016.00739 (PMC4882417; doi:10.3389/fpsyg.2016.00739)
Supplement: Supplementary file 1 [file Table1.pdf]

Participant	Condition	Deviants rejected / total	Standards rejected / total	Rejection rate<br>Deviant / standard in %
No 20	Misunderstanding<br>deviant /ro͡ʊsn̩/ 'roses'	9 / 60	25 /120	15 / 20.83
	deviant /ro͡asn̩/ 'journeys'	18 / 60	42 /120	30 / 35
No 2	Incomprehension prime	9 / 60	25 /120	15 / 20.83
	Incomprehension neutral	20 / 60	37 /120	33.33 / 30.83
No 14	Incomprehension prime	29 / 60	62 /120	48.33 / 51.67
	Incomprehension neutral	39 / 60	68 /120	65 / 56.67
No 20	Potential comprehension prime	21 /60	32 /120	35 / 26.67
	Potential comprehension neutral	21 / 60	27 /120	35 / 22.5

Exact rejection numbers for excluded participants after artifact scanning
