# Supplementary material for: Dialect Variation Influences the Phonological and Lexical-Semantic Word Processing in Sentences. Electrophysiological Evidence from a Cross-Dialectal Comprehension Study
Source: Front Psychol. 2016 May 27;7:739. doi: 10.3389/fpsyg.2016.00739 (PMC4882417; doi:10.3389/fpsyg.2016.00739)
Supplement: Supplementary file 2 [file Presentation1.pdf]

Diachronic development of the /o͡a/-diphthong in the Bavarian-Alemannic transition zone and Central Bavarian

In German, one of the most important isoglosses, which structure the Southern German dialect area, is the one between the Bavarian and the Alemannic dialects. Here, dialect classification maps show a transition zone in which phonological forms of both dialects interact with each other (cf. Wiesinger, 1983). Furthermore, this transition zone could be identified as an area of relatively high diachronic dynamics (cf. Lameli, 2013). Previous studies show a lexically gradual (word-by-word) change of the dialect phoneme corresponding to MHG[1] *ô* in words like *groß* 'big', *Rose* 'rose', *tot* 'dead' etc. in the Bavarian-Alemannic transition area (cf. Schmidt & Herrgen, 2011; Schmidt, 2010). Since irregularities in sound change are often a result of dialect contact, an analysis of the neighboring dialects might illuminate this phoneme change. The dialect phonemes corresponding to MHG *ô* and MHG *ei* have merged in BA. Both phonemes were pronounced identically like the falling diphthong /o͡a/, as historical data from the end of the 19th century show (cf. Schmidt et al., 2008ff). As a result of this merger, a number of homophonic lexemes has developed when a minimal pair can be formed only differing in the MHG corresponding segment *ô* and *ei*, e.g., /ro͡asn̩/ (Standard German *Rosen* 'roses' – *Reisen* 'journeys'), /bro͡at/ (Standard German *Brot* 'bread' – *breit* 'broad'). In this minimal pair condition, recent data tend to show variation in the former diphthong corresponding to MHG *ô* (cf. König, 1998; Eichinger, 2011; Rein, 2005). In these cases, the /o͡a/-diphthong is mostly replaced by an /o͡ʊ/-diphthong or an /oː/-monophthong. The /o͡a/-diphthong corresponding to MHG *ei* remains stable (e.g., /lo͡ap/ 'loaf', /ʃʊo͡af/ 'tail') as well as the same diphthong corresponding to MHG *ô*, when no minimal pair can be formed (e.g., /ʃto͡as/ 'stack', /ʃtro͡a/ 'straw'). In the Central Bavarian dialect area, the distinction of the phonemes in question has been maintained in the phonological system. Just like in BA, the dialect phoneme corresponding to MHG *ei* is pronounced like /o͡a/, but the one corresponding to MHG *ô* like /o͡ʊ/. Since the /o͡ʊ/-phoneme occurs in BA as well, namely as the dialect realization for MHG short *o*, this contact situation differs fundamentally from most language contact settings. The speech communities under investigation both possess the phonemes /o͡a/ and /o͡ʊ/. The crucial fact here concerns how the respective phonemes have been assigned to lexemes in the dialect areas. For this reason, the study focuses on the effects of the different phoneme to lexeme assignment on cross-dialectal comprehension caused by the merger of MHG *ô* and MHG *ei* in BA (see table 1).

Standard German	MHG phoneme	BA	CB	Interaction between BA (speaker) → CB (listener)
Laib 'loaf'	*ei*	/lo͡ap/	/lo͡ap/	understanding
Reisen 'journeys'	*ei*	/ro͡asn̩/	/ro͡asn̩/	understanding
Rosen 'roses'	*ô*	/ro͡asn̩/	/ro͡ʊsn̩/	misunderstanding as 'journeys'
Stoß 'stack'	*ô*	/ʃto͡as/	/ʃto͡ʊs/	incomprehension

The assignment of /o͡a/ and /o͡ʊ/ to lexemes in the Bavarian-Alemannic transition zone (BA) and Central Bavarian dialect area (CB)

[1] In German dialectology, historical reference systems such as Middle High German for vowels and West Germanic for consonants are commonly used as reference points for observable language developments in later language stages. These reference points provide the opportunity to compare recent dialectal phoneme systems with one another directly.
